# GABAergic Signaling Is Linked to a Hypermigratory Phenotype in Dendritic Cells Infected by *Toxoplasma gondii*

**DOI:** 10.1371/journal.ppat.1003051

**Published:** 2012-12-06

**Authors:** Jonas M. Fuks, Romanico B. G. Arrighi, Jessica M. Weidner, Suresh Kumar Mendu, Zhe Jin, Robert P. A. Wallin, Bence Rethi, Bryndis Birnir, Antonio Barragan

**Affiliations:** 1 Center for Infectious Medicine, Department of Medicine, Karolinska Institutet, Karolinska University Hospital Huddinge, Stockholm, Sweden; 2 Swedish Institute for Communicable Disease Control, Stockholm, Sweden; 3 Department of Neuroscience, Uppsala University, Uppsala, Sweden; 4 Department of Microbiology, Tumor and Cell Biology, Karolinska Institutet, Karolinska University Hospital Huddinge, Stockholm, Sweden; University of Pennsylvania, United States of America

## Abstract

During acute infection in human and animal hosts, the obligate intracellular protozoan *Toxoplasma gondii* infects a variety of cell types, including leukocytes. Poised to respond to invading pathogens, dendritic cells (DC) may also be exploited by *T. gondii* for spread in the infected host. Here, we report that human and mouse myeloid DC possess functional γ-aminobutyric acid (GABA) receptors and the machinery for GABA biosynthesis and secretion. Shortly after *T. gondii* infection (genotypes I, II and III), DC responded with enhanced GABA secretion *in vitro*. We demonstrate that GABA activates GABA_A_ receptor-mediated currents in *T. gondii*-infected DC, which exhibit a hypermigratory phenotype. Inhibition of GABA synthesis, transportation or GABA_A_ receptor blockade in *T. gondii*-infected DC resulted in impaired transmigration capacity, motility and chemotactic response to CCL19 *in vitro*. Moreover, exogenous GABA or supernatant from infected DC restored the migration of infected DC *in vitro*. In a mouse model of toxoplasmosis, adoptive transfer of infected DC pre-treated with GABAergic inhibitors reduced parasite dissemination and parasite loads in target organs, e.g. the central nervous system. Altogether, we provide evidence that GABAergic signaling modulates the migratory properties of DC and that *T. gondii* likely makes use of this pathway for dissemination. The findings unveil that GABA, the principal inhibitory neurotransmitter in the brain, has activation functions in the immune system that may be hijacked by intracellular pathogens.

## Author Summary

*Toxoplasma gondii* is an obligate intracellular protozoan parasite and an important food- and water-borne human and veterinary pathogen. Toxoplasmosis is normally self-limiting but severe manifestations occur upon congenital transmission to the developing fetus or during infection in immune-compromised individuals. *Toxoplasma* invades a variety of cell types and mounting evidence shows that certain white blood cells, e.g. dendritic cells, can shuttle parasites in the infected host by a Trojan horse type of mechanism. Dendritic cells are considered the gatekeepers of the immune system but can, paradoxically, also mediate dissemination of the parasite. Previous work has shown that *Toxoplasma* induces a hypermigratory state in dendritic cells when they become infected. Here, we show that, shortly after infection by the parasite, dendritic cells start secreting γ-aminobutyric acid (GABA), also known as the major inhibitory neurotransmitter in the brain. We show that dendritic cells express GABA receptors, as well as the machinery to synthesize and transport GABA. When GABA synthesis, transport or receptor function was inhibited, the migration of infected dendritic cells was impaired. In a mouse model of toxoplasmosis, treatment of infected dendritic cells with GABA inhibitors resulted in reduced propagation of the parasite. This study establishes that GABAergic signaling modulates the migratory properties of dendritic cells and that the intracellular pathogen *Toxoplasma gondii* sequesters the GABAergic signaling of dendritic cells to assure propagation.

## Introduction

*Toxoplasma gondii* is an obligate intracellular parasite that infects warm-blooded vertebrates. It infects approximately 25% of the global human population [1]. Initial infection occurs orally or congenitally, whereby the formed tachyzoite stages disseminate widely in the organism. Although principally asymptomatic in humans, infection can cause severe neurological complications in immune-compromised individuals, disseminated congenital infections in the developing fetus, and ocular manifestations in otherwise healthy individuals [1]. *T. gondii* enters host cells by active penetration, a rapid process that is dependent on the actin-myosin cytoskeleton of the parasite, and does not rely on the host cell machinery for uptake [2]. *T. gondii* can invade and multiply inside any nucleated cell type, including blood leukocytes, and a preference to infect myeloid leukocytes *in vitro* has been reported [3]. Following primary infection, *T. gondii* strikes a fine balance between eliciting an effective immune response and establishing a silent, life-long infection [4]–[6]. Acute infection triggers a robust Th1 polarized immune response with efficient activation of antigen presenting cells, including dendritic cells (DC) [7], [8].

DC are a fundamental component of the immune response but also a putative gate to immune evasion and persistence for pathogens [9]. DC serve as sensors in peripheral tissues that allow processing and presentation of antigens for initiation of adaptive immune responses and pathogen clearance. The mechanisms underlying DC migration are complex and the molecular traffic signals that govern DC migration are not fully understood [10]. One of the hallmarks of mature DC is the expression of the C-C chemokine receptor 7 (CCR7). Binding to its ligands (CCL19 and CCL21) guides the migrating cells to the lymph nodes where adaptive immune response is initiated [11]. In order to avoid clearance by the immune system, intracellular parasites, bacteria, fungi and virus have evolved diverse strategies to subvert this central function of DC [9], [12].

Mounting evidence indicates that DC play a pivotal role during *T. gondii* infection as mediators of essential immune responses [8], [13] and as parasite carriers that facilitate the dissemination of the infection [14]–[17]. In this context, *T. gondii* induces a hypermotility state in infected DC that contributes to parasite dissemination *in vivo* [14], [15]. Interestingly, this strategy for dissemination appears to be conserved among other members of the Apicomplexan parasite family, e.g. *Neospora caninum* [18]. Yet, the molecular mechanism controlling the parasite-induced hypermigratory phenotype in DC remains unknown. Given its characteristics, i.e. random directional hypermotility in absence of chemotactic cues, alternative/non-classical pathways are likely to be involved [4].

γ-aminobutyric acid (GABA) is one of the major neurotransmitters in the CNS [19], acting via activation of GABA_A_ receptors [20] and to a lesser extent GABA_B_ receptors [21]. GABA is shuttled in and out of cells via GABA transporters (GAT) of the solute carrier family 6 [22]. GABAergic cells synthesize GABA via glutamate decarboxylases (GAD) [23]. In contrast to its role as an inhibitory neurotransmitter, GABA plays an excitatory role during neuronal development [24], [25]. In fact, mounting evidence indicates that neurotransmitters, including GABA, have a motogenic function and participate outside the CNS in diverse functions including cell migration, immunomodulation, and metastasis [26], [27]. GABA, its synthesis enzymes GAD, GABA receptors and transporters have been found in a variety of tissues outside the CNS, such as the pancreatic islets and testes [28], [29].

Using *in vitro* models and *in vivo* bioluminescence imaging (BLI) in a mouse model of toxoplasmosis, we demonstrate that DC are GABAergic cells and that GABA modulates the hypermigratory phenotype observed in *Toxoplasma*-infected DC. During *in vivo* infections, the GABAergic system of infected DC is likely used to facilitate parasite dissemination.

## Results

### Mouse and human DC secrete GABA upon infection with *T. gondii*

**Figure 1:**
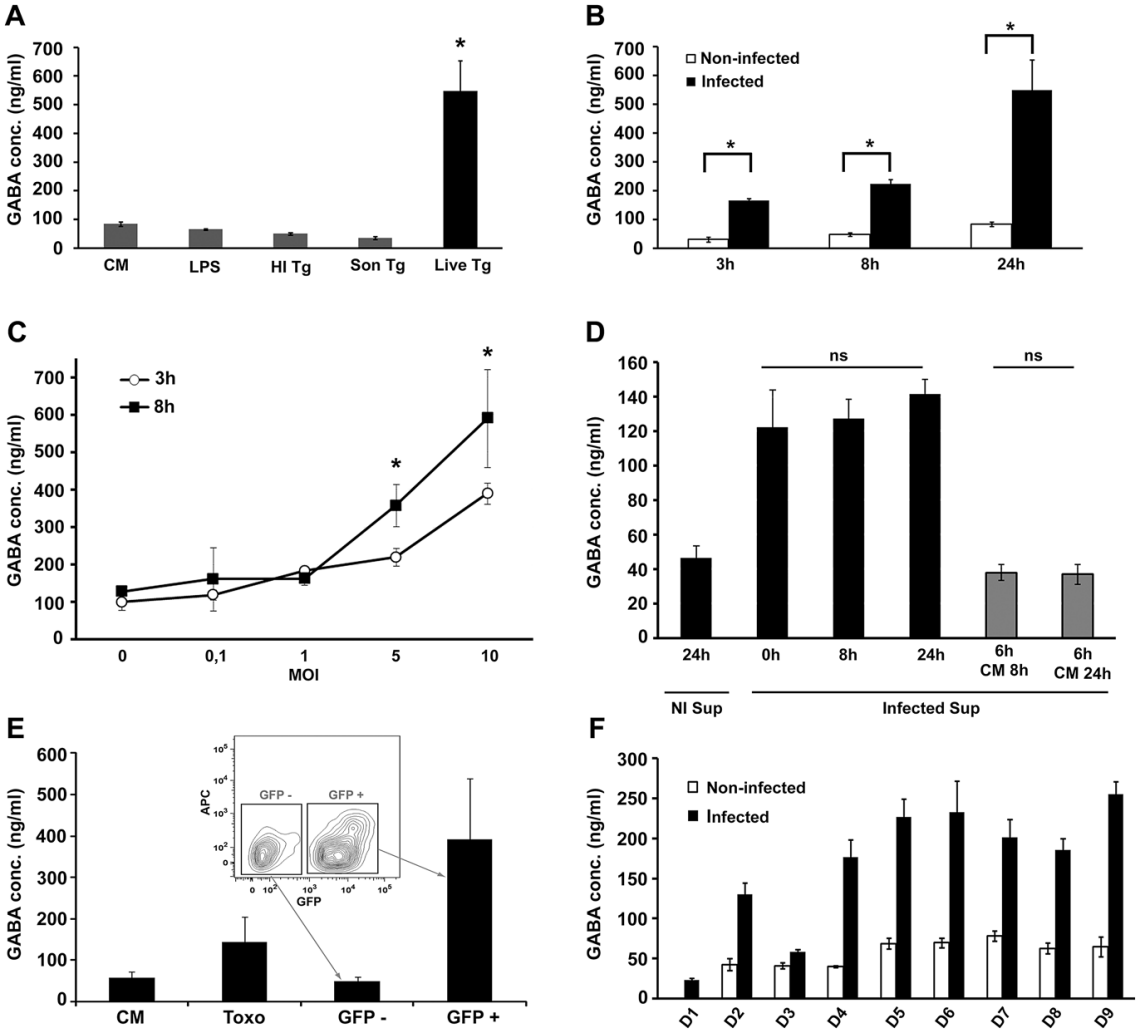
DC demonstrate GABAergic properties upon *T. gondii* infection. (**A**) Live *Toxoplasma* infection of mouse DC enhances the release of GABA. DC were incubated for 24 h with complete medium (CM), lipopolysaccharide (LPS, 100 ng/ml), heat inactivated *T. gondii* PTGluc tachyzoites (HI Tg), sonicated PTGluc tachyzoites (Son Tg) or freshly egressed PTGluc tachyzoites (Live Tg, MOI 1). GABA in the supernatant was quantitatively determined by ELISA as described under Materials and Methods. Values represent means (±SEM) from two experiments performed in quadruplicate. (*) indicates significant differences (P = 0.0045, Mann Whitney U-test). (**B**) Mouse DC GABA release occurs rapidly following *Toxoplasma* infection. DC were challenged with PTGluc tachyzoites (MOI 1) and GABA was quantitatively determined at indicated time points as described under Materials and Methods. Values represent means (±SEM) from two experiments performed in quadruplicate. (*) indicates significant increase in GABA production compared to non-infected DC (P<0.0001, GLM ANOVA, Tukeys Pairwise comparison). (**C**) Effect of multiplicity of infection (MOI) on GABA secretion from DC over time. Mouse DC were challenged with PTGluc tachyzoites at indicated MOI and GABA concentration in the supernatant was quantitatively determined after 3 h or 8 h by ELISA. Values represent means (±SEM) from one representative experiment performed in triplicate. Performed three times with similar result. (*) indicates significant differences (P<0.05, Student's t test). (**D**) Supernatants from infected DC do not induce secretion of GABA. Graph shows GABA concentrations after exposure of 10^6^ non-infected mouse DC to supernatants from infected DC (PTGluc, MOI 1, 24 h, 700 µl). “NI Sup 24 h” indicates GABA ELISA readout from DC in CM for 24 h; “I sup 0-8-24 h” indicate GABA concentrations after exposure of non-infected DC to supernatant from infected DC for 0, 8 and 24 h respectively. “I Sup 6 h + CM 8–24 h” indicate that non-infected DC were exposed to supernatant from infected DC for 6 h; the supernatant was then washed away and substituted by CM for 8 and 24 h respectively before assessment of GABA concentrations. Values represent means (±SEM) from one representative experiment performed in triplicate. Performed twice with similar result. ns: non-significant differences (P>0,05, Student's t-test). (**E**) Infected (GFP^+^) DC exhibit elevated GABA secretion but not uninfected (GFP^−^) by-stander DC. DC were challenged with freshly egressed GFP-expressing tachyzoites (PTGluc, MOI 1) for 6 h followed by fluorescence-activated cell sorting as indicated under Materials and Methods. For all conditions, cells were washed, replated in CM and secreted GABA (1×10^6^ DC/condition) was quantified after 16 h incubation as indicated under Materials and Methods. “CM” indicates DC in complete medium, “Toxo” indicates DC challenged with *T. gondii* that were subsequently subjected to cell sorting, illustrated by contour plot. Values represent means (±SD) from three independent experiments performed in duplicate. Significant differences were observed between GFP^−^ and GFP^+^ cell populations (P<0.0001, Paired t-test). (**F**) Human monocyte-derived DC are GABAergic. DC were infected with PTGluc tachyzoites (MOI 1) and GABA in the supernatant was analyzed after 24 h as described under Materials and Methods. Values represent means (±SEM) from each individual donor (n = 9) performed in quadruplicate.

To address the GABAergic response of mouse DC upon infection, GABA was quantified in the cell supernatant. Challenge of DC with freshly egressed *T. gondii* tachyzoites led to a significant increase of GABA in the supernatant, while heat inactivated parasites, parasite lysate or LPS did not increase GABA secretion relative to non-infected DC (Figure 1A). Moreover, secretion of GABA from DC challenged with freshly egressed tachyzoites rapidly increased over time, even prior to parasite replication, and augmented over 24 h (Figure 1B). In contrast, the GABA-precursor glutamate exhibited a modest transient increase in the supernatant following infection, which was redundant by 24 h (Figure S1). We next assessed if GABA secretion was induced in infected DC or uninfected bystander DC. GABA secretion rapidly augmented with MOI over time (Figure 1C) and supernatants from infected DC did not induce significant GABA secretion in DC (Figure 1D). Moreover, fluorescence-activated cell sorting of DC populations challenged with GFP-expressing *T. gondii* showed that GABA secretion occurred essentially in GFP^+^ cells (Figure 1E). Altogether, this shows that the observed elevation of GABA secretion emanates from infected DC and that GABA secretion of by-stander DC and DC in complete medium (CM) are similar. Next, 9 human donors were assessed. Monocyte-derived DC from all donors responded with increased amounts of GABA upon *T. gondii* infection and variability in the secreted levels of GABA was observed among the donors (Figure 1F). Monocytes challenged with *T. gondii* also exhibited an increase in GABA secretion (Figure S2A). Representative strains from the three predominant *T. gondii* genotypes (I, II and III) induced GABA secretion in infected DC (Figure S3). We conclude that upon *Toxoplasma*-infection, mouse and human myeloid DC exhibit elevated levels of GABA secretion.

### Mouse and human DC express functional GABA_A_ receptors

**Figure 2:**
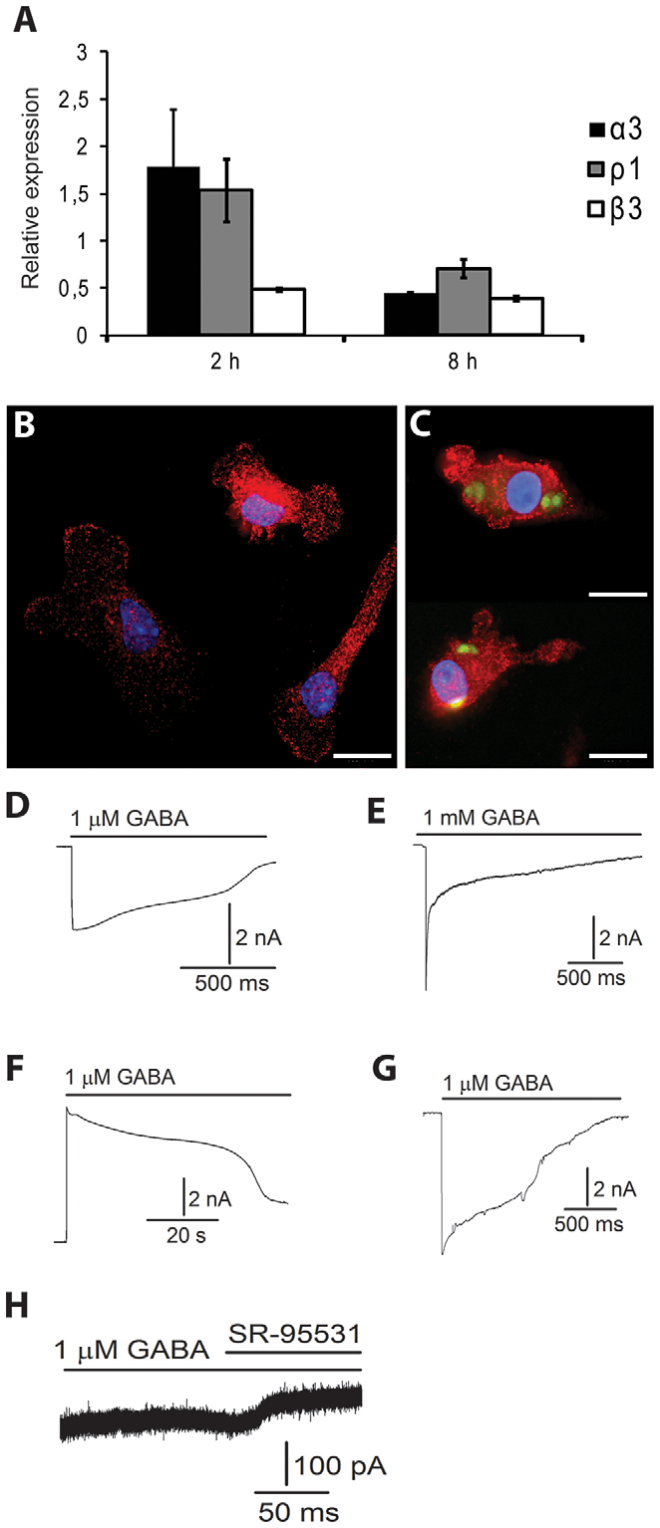
DC express GABA_A_ receptor subunits and GABA activates GABA_A_ receptor-mediated currents in DC. (**A**) GABA_A_ α_3_, β_3_ and ρ_1_ subunit expression is modulated in DC upon challenge with *T. gondii*. Mouse DC were incubated with PTGluc tachyzoites (MOI 3) or CM for 2 h or 8 h. RNA was extracted and cDNA synthesized as detailed in Materials and Methods. Following RT-qPCR, GABA_A_R α_3_, β_3_ and ρ_1_ subunit expression was determined using the ΔΔCt method, with GAPDH as the reference gene, and non-infected DC GABA_A_R α_3_, β_3_ and ρ_1_ expression as the calibrator, respectively. Y-axis indicates relative expression against non-infected DC calibrator. Values represent means (±SEM) from two or three independent experiments performed in triplicate. (**B–C**) Immunocytochemistry of non-infected DC (B) and DC challenged with GFP-expressing *T. gondii* tachyzoites (green) (C) stained with GABA_A_ receptor β_3_ subunit monoclonal antibody (red) and DAPI (blue). In C, upper micrograph shows replicating tachyzoites (green) and lower micrograph shows tachyzoites (green) shortly after invasion. Stainings were performed as indicated under Materials and Methods. Scale bar: 10 µm. (**D–H**) GABA activates GABA_A_ receptor-mediated currents in DC. Patch-clamping of *Toxoplasma*-infected and non-infected DC was performed as indicated under Materials and Methods. Whole-cells currents were evoked by application of 1 µM or 1 mM GABA to mouse DC (n = 7; D, E, F) or human DC (n = 7; G, H). In symmetrical chloride solution the currents were inward at negative potentials (D, E, G, H; −80 mV), outward at positive potentials (F; +40 mV) and blocked by SR-95531, a GABA_A_ competitive antagonist (H). D, E, F and H show results from infected (12 h) DC whereas G was recorded from a non-infected DC.

In an effort to ascertain which GABA_A_ receptor subunits are expressed in mouse DC, we screened the 19 subunits expression profiles in mouse DC and astrocytes. We detected GABA_A_R α_3,_ α_5_, β_1_, β_3,_ and ρ_1_ subunit transcripts in DC, whilst 12 different subunits were detected in primary astrocytes (Table 1, Table S1 for primer sequences). We decided to quantify differential gene transcription in mouse DC following *T. gondii* infection using α_3,_ β_3_ and ρ_1,_ the most strongly expressed subunits in non-infected mouse DC (Table 1). The transcript level analysis, using template from infected DC and non-infected DC, showed an up-regulation for the α_3_ and ρ_1_ transcripts after 2 h infection and down-regulation by 8 h. A down-regulation was observed for the β_3_ subunit at both time-points (Figure 2A). In addition, immunocytochemical stainings indicated expression of the β_3_ subunit in DC in CM (Figure 2B) and in *Toxoplasma*-infected DC (Figure 2C). In DC suspensions challenged with *T. gondii*, similar staining patterns were observed in infected and non-infected DC (Figure S4).

**Table 1:** Characterization of GABA_A_ receptor subunit gene expression in murine bone marrow-derived DC related to primary murine astrocytes.

Cell type	α1	α2	α3	α4	α5	α6	β1	β2	β3	γ1	γ2	γ3	δ	θ	ε	π	ρ1	ρ2	ρ3
**DC**			**x** a		**x**		**x**		**x** a								**x** a		
**Astrocytes**	**x**	**x**	**x**		**x**		**x**	**x**	**x**			**x**		**x**		**x**	**x**		**x**

A PCR screen of the 19 GABA_A_ subunits was performed using mouse DC cDNA and mouse astrocyte cDNA as a positive control as indicated under Materials and Methods.

a The transcripts of GABA_A_ receptor subunits α3, β3, and ρ1 were consistently quantified, and mean Ct values from non-infected DC cDNA were 37,0; 30,8; and 34,9 respectively. The transcripts of subunits α5 and β1 could not be consistently quantified.

We next examined functional expression of the GABA_A_ channels in DC using the whole-cell patch-clamp technique. We recorded currents from human and mouse DC infected for 12 h with *T. gondii* (Figure 2D, E, F, H) and non-infected DC (Figure 2G). At a negative holding potential (- 80 mV) in symmetrical chloride solutions, 1 µM GABA application to the cells resulted in an inward current that ranged widely in magnitude. In mouse and human DC the peak-current value ranged from −9 pA to −9.9 nA (n = 7) and −38 pA to −7.7 nA (n = 7), respectively. The currents reversed at positive holding potential (Figure 2F, +40 mV) and were inhibited by the GABA_A_ competitive antagonist SR-95531 (Figure 2H). We conclude that human and mouse myeloid DC express functional GABA_A_ receptors and that GABA can induce membrane currents in *Toxoplasma*-infected DC.

### Targeting GABA synthesis and transport reduces transmigration of infected DC *in vitro*

**Figure 3:**
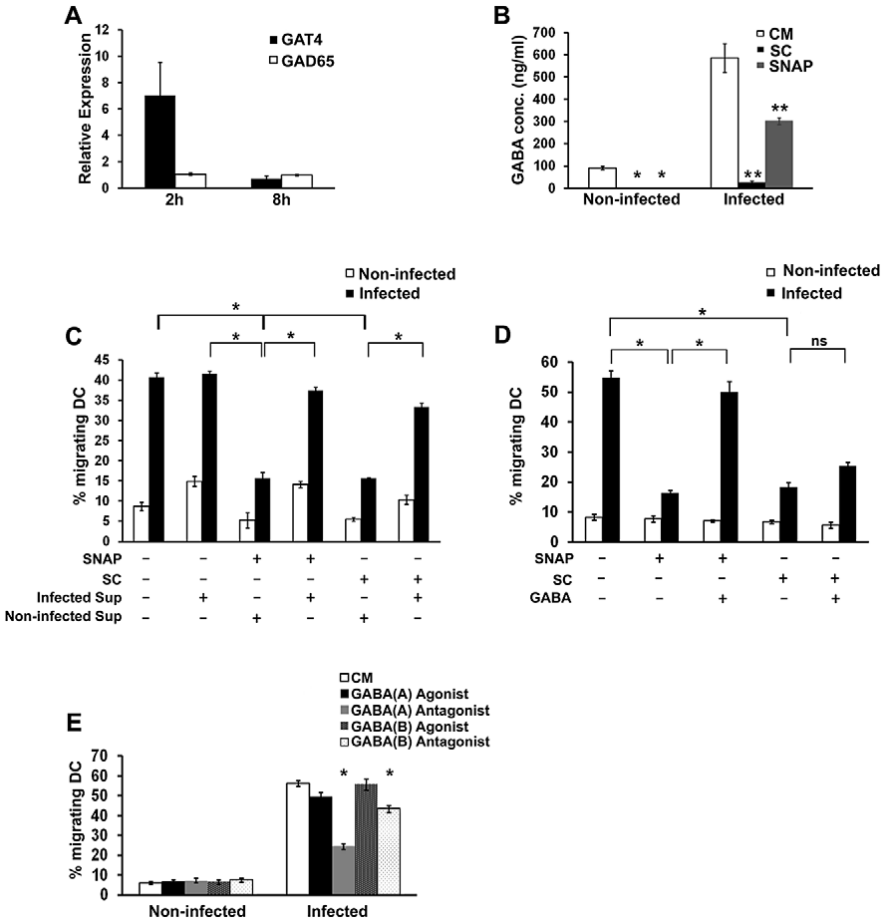
The GABAergic system is modulated in *Toxoplasma*-infected DC to facilitate hypermotility. (**A**) GAT4 expression is up-regulated following *Toxoplasma* infection whilst GAD65 is expressed in both non-infected and *Toxoplasma*-infected mouse DC. DC were incubated with PTGluc tachyzoites (MOI 3) or CM for 2 h or 8 h. RNA was extracted and cDNA synthesized as detailed in Materials and Methods. Following RT-qPCR, GAT4 and GAD65 differential expression was determined using the ΔΔCt method, with GAPDH as the reference gene, and non-infected DC GAT4 and GAD65 expression as the calibrator, respectively. Values represent means (±SEM) from two or three independent experiments performed in triplicate. (**B**) GAD and GAT4 antagonists inhibit GABA secretion in DC. Mouse DC were incubated with PTGluc tachyzoites (MOI 1) for 24 h under different conditions. CM indicates complete medium; SC (Semicarbazide, GAD inhibitor, 50 µM); SNAP (SNAP-5114, GAT4 inhibitor, 50 µM). GABA levels were analyzed using a GABA ELISA kit as described in Materials and Methods. Values represent means (±SEM) from two independent experiments performed in quadruplicate. (*) indicates a significant decrease in GABA production compared to non-infected DC in CM (P<0.002, Mann Whitney U-Test). (**) indicates a significant decrease in GABA production compared to untreated *Toxoplasma*-infected DC (P<0.008, Mann Whitney U-test). (**C**) The inhibitory effects of GAT4 and GAD65 antagonists on *Toxoplasma*-infected mouse DC transmigration can be restored following the addition of GABA enriched supernatant from infected DC. DC were infected for 6 h with PTGluc tachyzoites (MOI 3) or PTGluc tachyzoites plus SNAP (50 µM) or SC (50 µM) as described in Materials and Methods. DC were then transferred to transwells, and allowed to transmigrate in the presence of CM, SNAP (50 µM), SC (50 µM), or SNAP/SC plus supernatant from infected DC. Cell migration was determined using a neubauer hemocytometer. Values represent means (±SEM) from two independent experiments done in triplicate. (*) indicates significant differences (P<0.01, One-way ANOVA). (**D**) The inhibitory effects of the GAT4 antagonist SNAP on infected DC transmigration can be fully restored following the addition of exogenous GABA. Mouse DC were infected for 6 h with PTGluc tachyzoites (MOI 3) or PTGluc tachyzoites plus SNAP (50 µM) or SC (50 µM) as described in Materials and Methods. DC were then transferred to transwells, and allowed to transmigrate in the presence of CM, SNAP (50 µM), SC (50 µM), SNAP plus GABA (50 µM and 0.5 µM respectively), or SC plus GABA (50 µM and 0.5 µM respectively). Cell migration was determined using a neubauer hemocytometer. Values represent means (±SEM) from two independent experiments done in triplicate. (*) indicates significant differences (P<0.01, One-way ANOVA). ns: non-significant. (**E**) *Toxoplasma*-induced DC transmigration is partially inhibited by GABA_A_ and GABA_B_ receptor inhibitors. Mouse DC were infected for 6 h with PTGluc tachyzoites (MOI 3) or PTGluc tachyzoites plus Muscimol (GABA_A_ Agonist– 300 µM), Bicuculline (GABA_A_ Antagonist – 50 µM), Baclofen (GABA_B_ Agonist – 500 µM), or CGP35348 (GABA_B_ Antagonist –500 µM) (as described in Materials and Methods). DC were then transferred to transwells, and allowed to transmigrate in the presence of complete medium (CM), Muscimol (GABA_A_ Agonist– 300 µM), Bicuculline (GABA_A_ Antagonist – 50 µM), Baclofen (GABA_B_ Agonist – 500 µM), or CGP35348 (GABA_B_ Antagonist –500 µM). Cell migration was determined using a neubauer hemocytometer. Values represent means (±SEM) from two independent experiments done in triplicate. (*) indicates significant differences for GABA_A_ receptor antagonist and GABA_B_ receptor antagonist (P<0.0001 and P<0.001 respectively, 2-way ANOVA).

To investigate the effects of *Toxoplasma* infection on the GABAergic system, expression levels of the GABA transporter GAT4 and the GABA synthesizing enzymes, GAD65 and GAD67, were assessed in DC. A rapid induction of GAT4 transcription was observed shortly after infection (Figure 3A). In contrast, expression of GAD65 was detected in both non-infected and infected DC at similar levels (Figure 3A), whereas GAD67 expression was not detectable in either group (data not shown). Moreover, addition of GAD inhibitor (SC) and GAT4 inhibitor (SNAP) to infected DC nearly abolished or significantly reduced, respectively, the secreted levels of GABA in the supernatant (Figure 3B). Inhibitor treatments did not significantly affect intracellular parasite replication *in vitro* (Figure S5) or the GABA signal detected in complete medium containing extracellular parasites (Figure S6). We next assessed the impact of the GABAergic inhibitors on the transmigration of infected DC. Both GAT4 (SNAP) and GAD inhibition (SC) had a significant inhibitory effect on the transmigration of infected DC, and transmigration was significantly restored following incubation of the inhibitor-treated cells in supernatant from infected DC cultures (Figure 3C). In contrast, monocytes did not exhibit this migratory phenotype observed in human and murine DC [14] but GABAergic inhibition significantly reduced the transmigration of non-infected monocytes (Figure S2B). Furthermore, the transmigration phenotype of DC was either fully (SNAP, GAT4 inhibitor) or partially (SC, GAD inhibitor) restored after addition of exogenous GABA (Figure 3D). In line with this, GABA_A_ receptor antagonist, and to a lesser extent GABA_B_ receptor antagonist, significantly reduced transmigration of infected DC (Figure 3E). Notably, GABA_A_ and GABA_B_ receptor agonists did not enhance transmigration of non-infected or infected DC. Thus, GABA *per se* was not sufficient to induce transmigration of non-infected DC but could restore transmigration in infected DC impaired in GABA production or transportation. Altogether, these data implicate GABA synthesis, transportation and receptor activity in parasite-induced transmigration of DC *in vitro*.

### GABAergic signaling modulates motility and chemotaxis of infected DC *in vitro*

**Figure 4:**
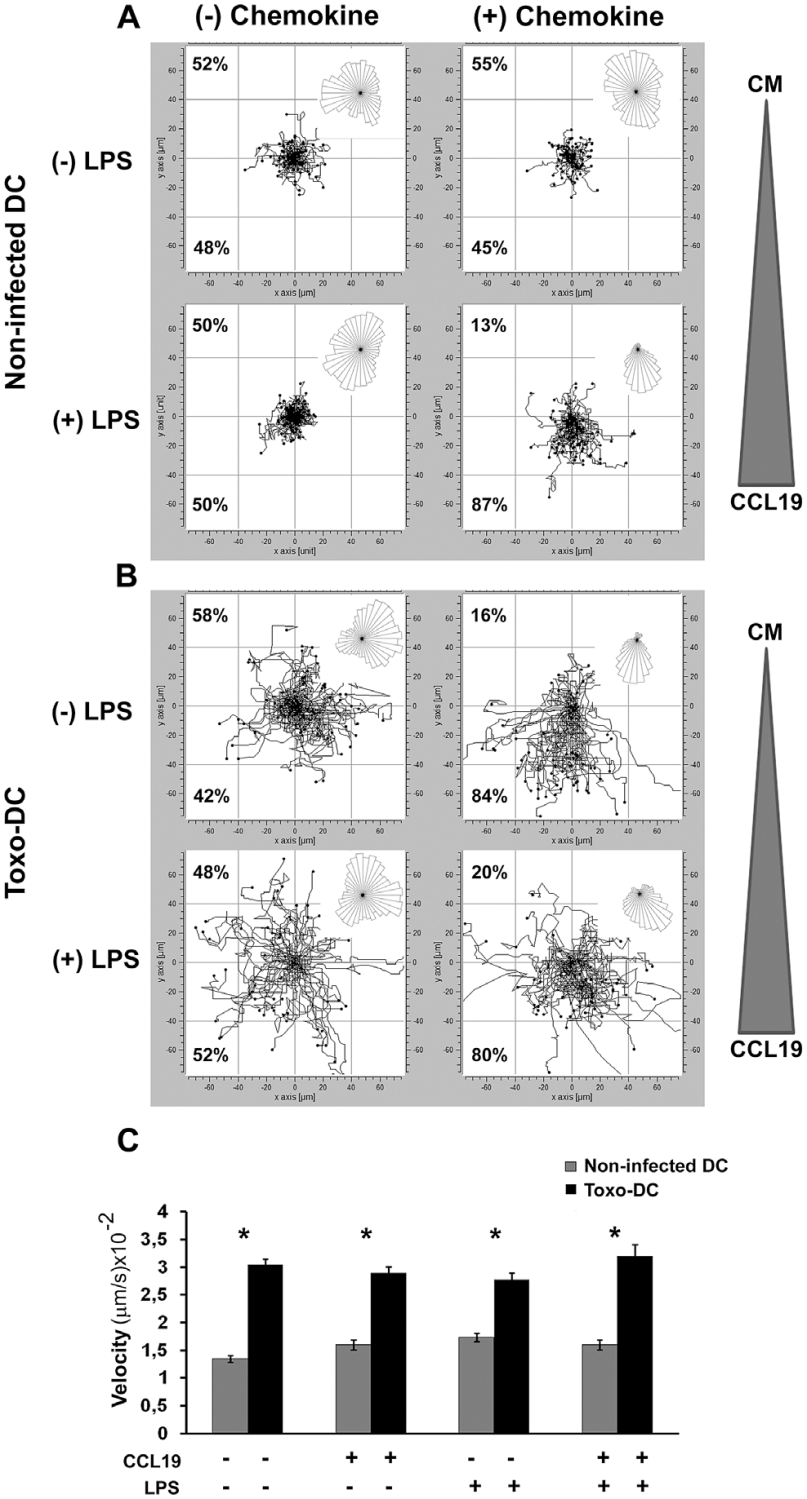
*Toxoplasma*-infected mouse DC exhibit hypermotility and chemotaxis. (**A**) Non-infected mouse DC or (**B**) *Toxoplasma*-infected mouse DC (PTGluc, MOI 1/Toxo-DC) were incubated ± LPS (200 ng/ml). After 24 h, cells were collected and placed in a chemotaxis chamber ± CCL19 gradient and tracked for 60 min as described under Materials and Methods. The tracking data presented is a composite of two independent experiments (n = 60 cells). The overall directionality of migration is depicted in the rose-diagram in the upper right corner of each single track summary. Percentages represent the proportion of cells migrating towards or away from chemoattractant or complete medium (CM). For the condition (+) chemokine, the triangle indicates the placement of the CCL19 chemokine gradient in each chemotaxis chamber. For (−) chemokine, CM was added to both chambers. (**C**) Velocity analysis of non-infected mouse DC or *Toxoplasma*-infected mouse DC in presence or absence of the chemokine CCL19. Bars indicate velocity (+SEM) from two independent experiments. (*) indicate significant differences (P<0.001, Mann Whitney U-test).

To determine whether the GABAergic system also affected DC motility and chemotaxis, infected and non-infected DC were allowed to migrate along a concentration gradient of CCL19 in a chemotaxis chamber system. Non-infected DC exhibited a low level of random directional motility in absence or presence of chemokine and LPS-stimulation of non-infected DC resulted in a distinct directional migration towards CCL19 (Figure 4A). In contrast, *Toxoplasma*-infected DC exhibited a dramatically enhanced random directional motility in absence of chemokine (Figure 4B), with a significant increase in velocity compared to non-infected DC (Figure 4C). Interestingly, directionality towards CCL19 was observed for infected DC, similar to that observed upon LPS maturation (Figure 4B). It is also notable that *Toxoplasma*-infected DC ((−) chemokine, Fig. 4 B) outranged LPS-matured non-infected DC ((+) chemokine, Figure 4A) in migrated distances and velocity (Figure 4A, B, C). We conclude that *Toxoplasma*-infected DC exhibit a hypermigratory phenotype *in vitro* and that hypermotile *Toxoplasma*-infected DC maintain the ability to chemotax *in vitro*.

**Figure 5:**
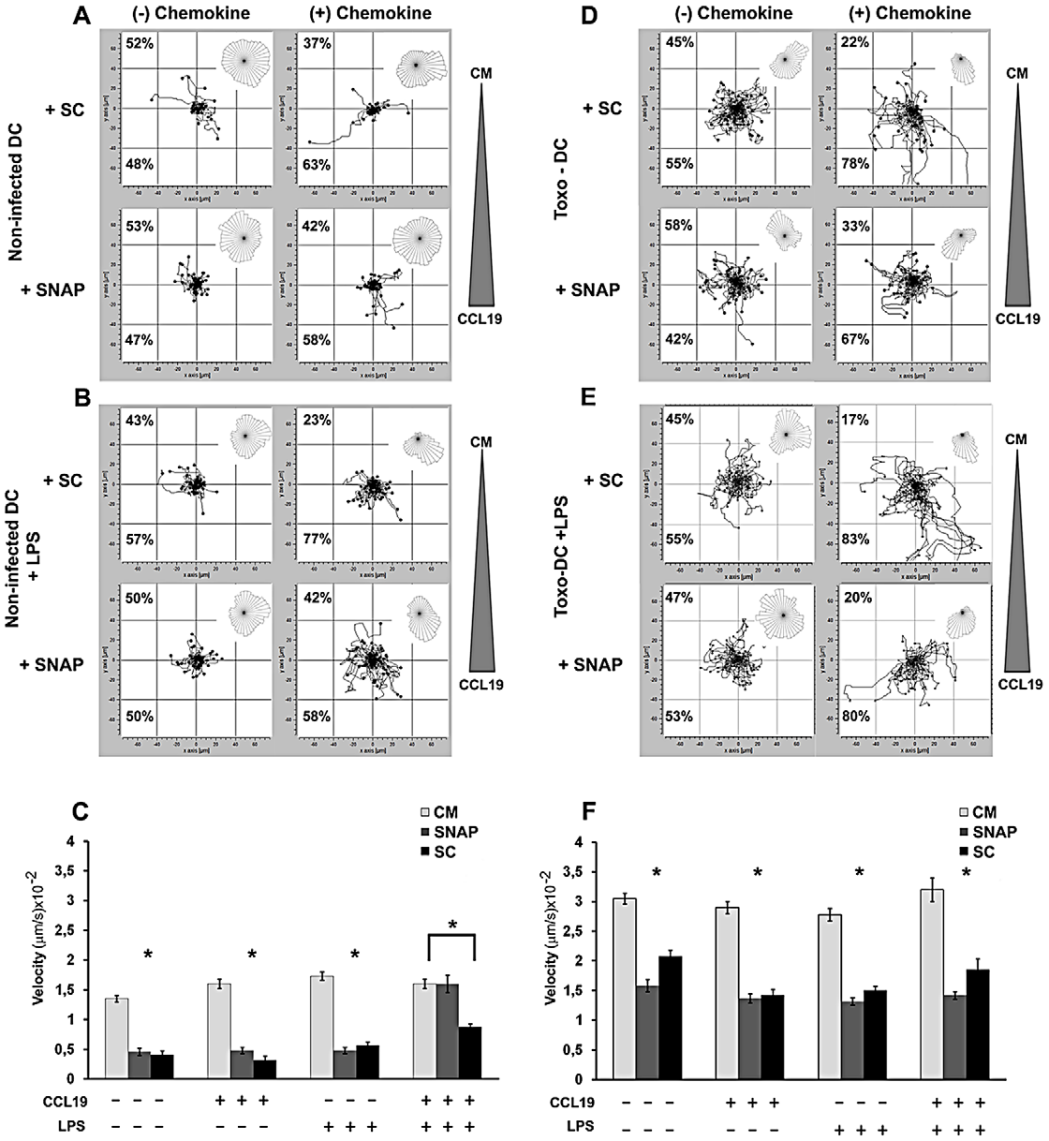
DC motility and chemotaxis are affected by the GABAergic system. (**A, B**) Non-infected mouse DC or (D, E) *Toxoplasma*-infected mouse DC (PTGluc, MOI 1/Toxo-DC) were treated with GAD inhibitor (SC, Semicarbazide, 50 µM) or GAT4 inhibitor (SNAP, SNAP5114, 50 µM) ± LPS (200 ng/ml). After 24 h, cells were collected and placed in a chemotaxis chamber ± CCL19 gradient and tracked for 60 min as described under Materials and Methods. The tracking data presented is a composite of one to two experiments performed in duplicate (n = 60 cells). The overall directionality of migration is depicted in the rose-diagram in the upper right corner of each single track summary. Percentages represent the proportion of cells migrating towards or away from chemoattractant or complete medium (CM). For the condition (+) chemokine, the triangle indicates the placement of the CCL19 chemokine gradient in each chemotaxis chamber. For (−) chemokine, CM was added to both chambers. (**C, F**) Velocity analysis of non-infected DC (A, B) and *Toxoplasma*-infected DC (D, E) in presence or absence of GABAergic inhibition (CM, complete medium; SC, GAD inhibitor; SNAP, GAT4 inhibitor) ± gradient with the chemokine CCL19. Bars indicate velocity (+SEM) from two independent experiments. (*) indicate significant differences (P<0.001, Mann Whitney U-test).

Next, we determined whether targeting the GABAergic system affected the migratory and chemotactic responsiveness of non-infected DC (Figure 5A, B) and *Toxoplasma*-infected DC (Figure 5D, E) *in vitro*. Overall, inhibition of GABA synthesis (SC, GAD inhibitor) or GABA transport (SNAP, GAT4 inhibitor) led to a significant decrease in the velocity and the accumulated distance covered by DC (Figure 5C, F). Interestingly, the ability to respond with directionality towards CCL19 was not abolished by inhibiting GABA transport or synthesis but, as a consequence of the reduction in velocity, the overall chemotactic response was diminished (Figure 5). No significant influence of a GABA gradient on the directionality of DC motility was observed for non-infected and infected DC (data not shown). In summary, present data show that inhibition of the GABAergic signaling system significantly reduces the velocity of infected DC *in vitro* and thereby the magnitude of the chemotactic response *in vitro*.

### Human and mouse DC exhibit upregulation of CCR7 upon *T. gondii* infection *in vitro*

**Figure 6:**
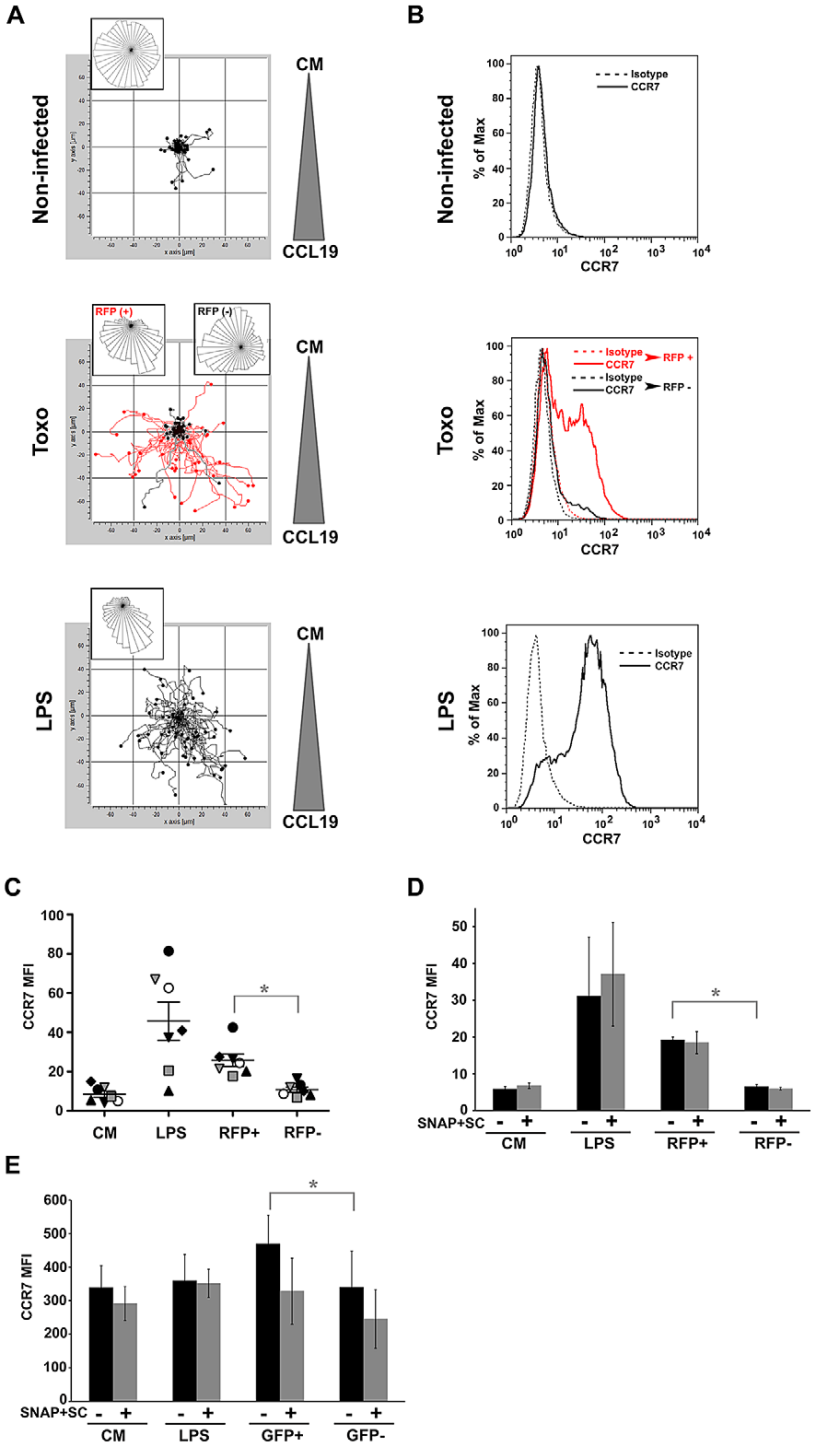
Chemotactic responses and relative expression of CCR7 in human and mouse DC. (**A**) Chemotaxis tracking analyses of human monocyte-derived DC. DC in complete medium (non-infected), DC challenged with freshly egressed RFP-expressing tachyzoites (Toxo, PRU-RFP, MOI 1, 24 h) or exposed to LPS (200 ng/ml, 24 h) were placed in chemotaxis chambers with a CCL19 gradient and tracked for 60 min as described under Materials and Methods. In the central panel, cells were separated into RFP^+^ (red tracks) and RFP^−^ (black tracks). The tracking data is a composite of two experiments performed in duplicate (n = 60 cells). The overall directionality of migration is depicted in the rose-diagrams of each single track summary. (**B**) Histograms show CCR7 expression of human monocyte-derived DC treated as in (A). CCR7 stainings (solid lines), isotype control stainings (dashed lines) and flow cytometry were performed as indicated under Materials and Methods. Displayed data are representative of 4 independent experiments and 7 donors. (**C**) Human monocyte-derived DC exhibit a significant upregulation of CCR7 upon *T. gondii* infection. Diagram shows, for each individual donor (n = 7), mean fluorescence intensity (MFI) analyzed by flow cytometry. Mean ± SEM is indicated for each group. CM: DC in complete medium; LPS: LPS-treated DC; DC challenged with *T. gondii* (PRU-RFP) were separated on RFP-positivity. (*) indicates significant difference (RFP^+^ vs. RFP^−^: *P*≤0.001, Paired *t*-test). (**D and E**) Effect of GABAergic inhibition on CCR7 expression in human DC (D) and mouse DC (E). Bar graphs show mean fluorescence intensity (MFI; ±SEM) analyzed by flow cytometry, from three independent experiments. Non-significant differences were observed in presence of GABAergic inhibitors (SC, SNAP) for the different conditions (*P*>0.05, Paired *t*-test). DC exhibited a significant upregulation of CCR7 upon *T. gondii* infection, indicated by asterisk (RFP^+^ vs. RFP^−^: *P = *0.0003 for human DC; GFP^+^ vs. GFP^−^: *P = *0.029 for mouse DC, Paired *t*-test).

We next assessed the relative expression of the CCL19 ligand CCR7 on human and mouse DC by flow cytometry. First, the chemotactic responses observed with mouse DC were confirmed using human monocyte-derived DC (Figure 6A). Additionally, monitoring of infected and uninfected DC in suspensions challenged with *T. gondii* showed that the chemotactic response occurred preferentially in the infected (RFP^+^) DC population (Figure 6A, central panel). In line with this result, DC challenged with *T. gondii* or treated with LPS exhibited a relatively higher expression of CCR7 compared to DC in complete medium (Figure 6B). The analyses of DC populations challenged with *T. gondii* showed that upregulation of CCR7 occurred essentially in infected (RFP^+^) DC (Figure 6B, central panel). An upregulation of CCR7 was consistently observed in infected DC from 7 different human donors (Figure 6C). For mouse DC, a small but significant upregulation of CCR7 was observed in infected DC (Figure 6E). In the presence of GABAergic inhibitors (SC, SNAP), overall non-significant effects on CCR7 expression were observed (Figure 6D and E). We also assessed the effects of GABAergic inhibitors (SC, SNAP) on the expression of co-stimulatory molecules and maturation. Overall, no distinct or modest effects were observed by GABAergic inhibition (Figure S7). Altogether, we conclude that upon *T. gondii*-infection, DC exhibit a relative up-regulation of CCR7 that is consistent with the observed chemotactic responses *in vitro*.

### GABAergic inhibition in adoptively transferred infected DC reduces the dissemination of *T. gondii in vivo*

**Figure 7:**
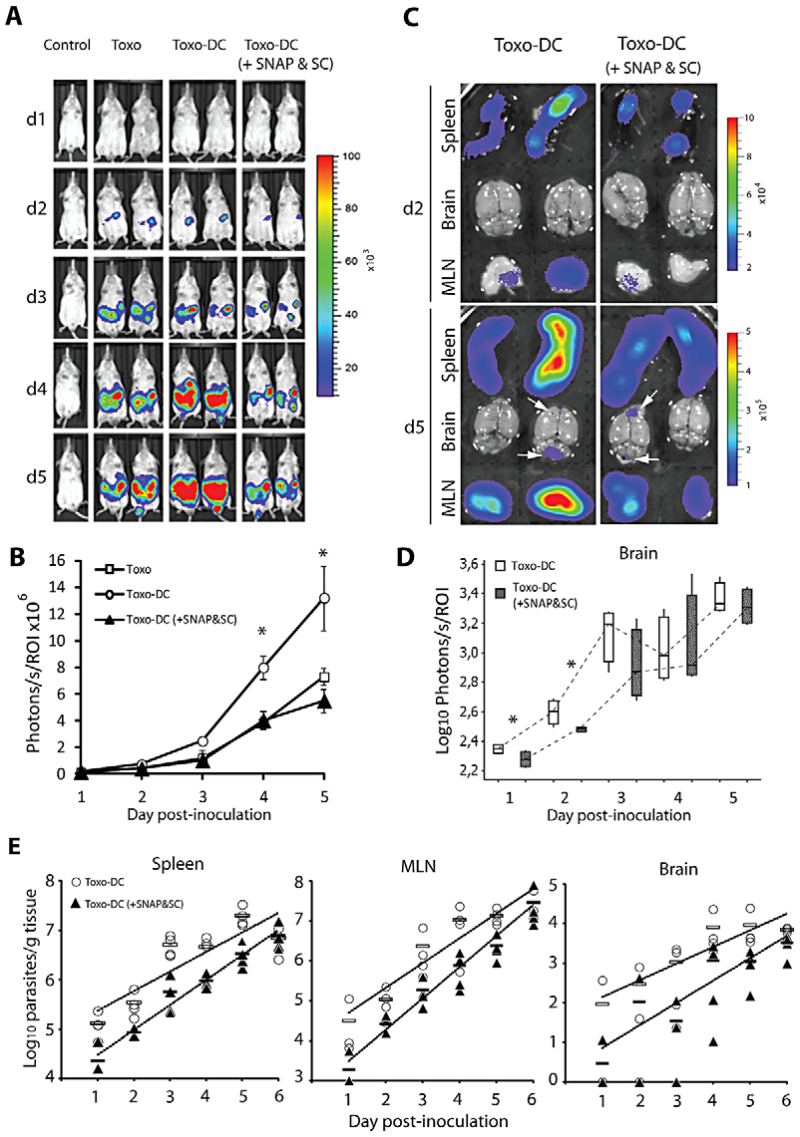
Decreased dissemination and parasite load after adoptive transfer of *Toxoplasma*-infected DC treated with GABAergic inhibitors. (**A**) BALB/c mice were challenged with 5×10^4^ cfu of freshly egressed PTGluc tachyzoites (Toxo), 5×10^4^ cfu of tachyzoite-infected DC (Toxo-DC) or 5×10^4^ cfu tachyzoite-infected DC treated with SNAP and SC (Toxo-DC + SNAP&SC). Photonic emissions were assessed by BLI on five consecutive days after inoculation of mice. Images show progression of infection and parasite biomass expansion in mice. Control shows non-infected mouse. Color scales indicate photo emission (photons/s/cm^2^/sr) during a 180 s exposure. Data are from one set of mice and representative of two independent experiments with three to five mice per group. (**B**) Total photon emission analysis from individual mice on days 1–5 post inoculation showed a decrease in parasite tissue burden in mice inoculated with GAD and GAT4 inhibitor-treated DC infected with *T. gondii* when compared with burdens from mice inoculated with untreated DC infected with *T. gondii*. Photonic emissions were assessed (photons/s/ROI) during a 180 s exposure. Data are from two independent experiments with 3–5 mice per group. Asterisks indicates a significant increase in photon emissions on day 4 and 5 post infection from mice in the “Toxo-DC” group compared to mice in the “Toxo” or “Toxo-DC (+SNAP&SC)” groups (P≤0.0004, GLM ANOVA, Tukeys Pairwise comparison). ROI – region of interest. (**C**) *Ex vivo* photonic emissions from spleen, MLN and brains of mice BALB/c mice challenged with 10^5^ cfu of tachyzoite-infected DC (Toxo-DC) or 10^5^ cfu tachyzoite-infected DC treated with SNAP and SC (Toxo-DC + SNAP&SC). Color scales indicate photonic emissions (photons/s/cm^2^/sr) during a 180 s exposure as indicated under Materials and Methods. Representative images from day 2 and day 5 post-inoculation are shown. White arrows (lower panel) indicate signal from the brain tissue. (**D**) Box-plot and whiskers graph represents the lower, upper quartiles, median and minimum–maximum of photonic emissions *ex vivo* from the brains of infected mice. The white bars indicate brains from mice infected with tachyzoite-infected DC (Toxo-DC) and the dark grey bars indicate brains from mice infected with tachyzoite-infected DC treated with SNAP and SC (Toxo-DC + SNAP&SC) (*n = *4/group/day). Non-significant differences were observed between the treated and untreated groups (P≥0.05; Kruskal Wallis test) with significant differences on days 1 and 2 (P≤0.05, Student's t test, indicated by asterisks). (**E**) Parasite load in spleen, MLN and brain on days 1–6 post inoculation quantified by plaquing assays as indicated under Materials and Methods. BALB/c mice were challenged with 10^5^ cfu of tachyzoite-infected DC (Toxo-DC, open circles) or 10^5^ cfu tachyzoite-infected DC treated with SNAP and SC (Toxo-DC + SNAP&SC, filled triangles). Mean parasite tissue load for each group is indicated (open and filled lines respectively) and dashed lines represent trendlines for the two groups (*n = *4/group/day). Significant differences in parasite load between groups were observed for spleen and brain (P≤0.022 and P≤0.02 respectively, Kruskal-Wallis test). Non-significant differences were observed in MLN (P≥0.05, Kruskal-Wallis test) with significant differences between groups on days 2, 4, 5, 6 (P≤0.05, Student's t-test).

Previously we have demonstrated that the adoptive transfer of *T. gondii*-infected DC leads to rapid dissemination of parasites as well as exacerbation of infection compared to infection with free parasites [14]. To assess whether GABAergic inhibition of *T. gondii*-infected DC had an impact on the aforementioned *in vivo* dissemination, mice were inoculated i.p. with freshly egressed luciferase-expressing tachyzoites or with tachyzoite-infected DC. Photonic emissions were measured by BLI daily for 5 days [30]. Infected DC were pretreated with a combination of inhibitors against GAD and GAT4 shown to have prolonged (24 h) inhibition on transmigration of DC *in vitro*. Interestingly, GABAergic inhibition of infected DC resulted in a significant reduction (∼2.8 fold) in total parasite photonic counts compared to non-treated infected DC by day 4 post infection (P = 0.0003, GLM ANOVA, Figure 7A and B). Furthermore, the photonic counts from the combination treated group were equivalent to levels observed during free tachyzoite infection (P>0.05, GLM ANOVA, Figure 7A and B). Analyses of adoptively transferred uninfected DC pretreated with GABAergic inhibitors showed similar numbers of treated and non-treated DC in the spleen and the peritoneum (Figure S8).

To determine the presence of parasites in different organs, photonic emissions were assessed *ex vivo* in the spleen, MLN and brain (Figure 7C). Special assessment of the brain showed significant differences in photonic emissions on days 1–2 with important variability between mice (Figure 7D). To quantify parasitic loads in target organs, plaquing assays were performed. Overall, higher parasitic loads were observed in mice challenged with non-treated infected DC compared to mice challenged with infected DC treated with GABAergic inhibitors (Figure 7E). Altogether, and in line with observations *in vitro*, this indicates that treatment of infected DC with GAD- and GAT4-inhibitor (SC, SNAP) results in a significant reduction in the dissemination of *T. gondii*, and subsequently a reduction of the parasitic loads during the course of infection in mice.

## Discussion

In the present study, we report that GABAergic signaling is closely linked to a hypermigratory phenotype in DC, which is induced by *T. gondii* infection [14]. Furthermore, we demonstrate that mouse and human myeloid DC possess functional GABA_A_ receptors and are capable of producing and secreting GABA. Interestingly, challenge of DC with *T. gondii* consistently resulted in a significant increase in the levels of extracellular GABA over time in mouse DC and in DC derived from different human donors. The secretion of GABA was not related to DC activation or maturation following exposure to LPS, parasite lysates, supernatants from infected DC or uptake of heat-inactivated parasites, but linked to the live infection by *T. gondii*. Our data indicates that DC secrete GABA as a consequence of infection by the parasite and that non-infected by-stander DC only provide a minor contribution to the total secreted amounts. Also, the absence of a distinct modulation on the secreted levels of the GABA-precursor glutamate is indicative of a selective effect on GABA synthesis and secretion.

We found that a determined subset of GABA_A_ receptors subunit genes was transcribed in mouse DC (α_3_, α_5_, β_1_, β_3_ and ρ_1_) in contrast to the broader expression in astrocytes. This finding is consistent with the concept that most GABA_A_ receptor pentamers are composed of at least two α- and two β-type subunits while the final subunit type may vary [31]. This also strongly suggests that genetic control plays a major role in the choice of transcribed subunit variants. In fact, such differential expression has been implicated in the changes in responsiveness and function of GABA_A_ receptors [32], [33]. Moreover, factors that affect GABAergic signaling, e.g. infection, may simultaneously induce up- and down-regulation of specific GABA_A_ subunits through epigenetic mechanisms [34].

Here, we demonstrate for the first time that GABA evokes GABA_A_ receptor-mediated currents in *T. gondii*-infected DC and in non-infected DC. While transcript levels of the α_3,_ β3 and ρ_1_ subunits were modulated upon infection, functional patch-clamping data indicates that GABA_A_ receptors are constitutively expressed in DC. In line with this, immunocytochemical analyses indicated expression of the GABA_A_ receptor β3 subunit in infected and non-infected DC. As individual receptor subunits do not necessarily reflect the number of functional receptor pentamers or combinations of pentamers that are expressed in a particular cell, it remains unknown how individual subunits relate to the receptor function in DC. Thus, further studies are needed to characterize and quantify the precise receptor subset composition of immune cells, and whether sensitivity to GABA is modulated upon infection.

Recently, human monocytes were shown to express the GABA_A_ β_2_ subunit and functional GABA_A_ receptors were described in a human myelomonocytic cell line (α_4_, β_2_, γ_1_ and δ subunits) [35]. In contrast to DC, monocytes do not exhibit enhanced transmigration upon *T. gondii*-infection *in vitro* [36]. Here, we report that monocytes respond with GABA secretion upon *T. gondii* infection. Altogether, this raises the questions if receptor activation through secreted GABA, e.g. an autocrine loop, is needed for migratory activation or if different subsets of receptors are expressed in DC compared to monocytes. Whether these intriguing phenotypic differences between monocytes and DC depend on GABA_A_ receptor expression levels, functional receptor subunit composition or capacity to rapidly secrete GABA awaits further investigation.

Induction of GABA secretion in infected DC was confirmed in strains from the three predominant genotypes of *T. gondii*, but it is unlikely that the maintenance and expression of GABA receptors and the GABAergic system in DC are exclusively a result of evolutionary pressure from *T. gondii*. Thus, additional functions for the GABAergic system in DC are likely to be discovered.

Mounting evidence indicates that *T. gondii* modulates the host's pathways for cell migration to facilitate its dissemination and establishment of a chronic infection. Our studies demonstrate that targeting the GABAergic machinery of host DC, i.e. GABA biosynthesis, transport, or ligand channel activation *in vitro* resulted in impaired ability to transmigrate, most prominently using inhibitors targeting host cell GAD and GAT4. Furthermore, the motility of treated infected DC was reduced to levels comparable to non-infected DC. In contrast, targeting the GABAergic system did not abrogate the ability of DC to respond with directionality in a CCL19 chemokine gradient but significantly reduced the speed and migrated distances of infected DC, thus reducing the chemotactic response *in vitro.* Further, in absence of a chemokine gradient, the motility and velocity of DC was reduced. This suggests that GABA and the GABAergic system may primarily interfere with the mechanisms of cell motility. In fact, GABA has been shown to promote the metastasis of cancer cells [37] and increase the velocity of human sperm motility via activation of GABA_A_ and GABA_B_ receptors [38]. Also, our *in vitro* chemotaxis data using GABA as a chemoattractant suggests that GABA alone is not sufficient to mitigate directional DC migration, irrespective of infection status or LPS maturation.

Notably, during *T. gondii* infections CCR7 and CCL19 are up-regulated [39], [40] and soluble factors can trigger upregulation of CCR7 in DC [41]. We have previously shown that *Toxoplasma*-induced hypermotility of DC *in vitro* occurs in absence of chemotactic cues and does not depend on CCR7-, CCR5- or MyD88-activation [14]. Here, we report that hypermotile *T. gondii*-infected human and mouse DC respond to chemotactic cues (CCL19) *in vitro* and significantly up-regulate CCR7. In neurons, reports indicate that GABAergic chemokinetic signaling cooperates with other chemotactic cues for embryonic cell migration [42]. In line with this, GABAergic inhibition has been shown to down-modulate the chemotactic responses of monocytes and neutrophils [35], [43]. Hypothetically, we propose that the two mechanisms tested here, GABA/GABA_A_ receptor-mediated hypermotility and CCR7-mediated chemotaxis, could be acting simultaneously or even synergistically in *Toxoplasma*-infected DC, thereby enhancing DC motility and potentiating the dissemination of parasites infecting DC. Also, possible synergistic effects with activation of multiple chemokine receptors or downstream signaling [14], [40], [44] need to be tested *in vivo* in future research, especially in the context of the remarkably efficient passage of the parasite across restrictive biological barriers.

Present data are consistent with the notion that *T. gondii* is able to subvert the regulation of host cell motility and exploits the host's natural pathways of cellular migration for parasite dissemination [4]. In line with the above, GABAergic inhibition in *T. gondii*-infected DC adoptively transferred to mice abolished the disseminatory advantage provided by ‘shuttling’ DC [14] and resulted in a reduced parasite burden over the course of infection in mice. Although the overall trend was that GABAergic inhibition yielded generally lower parasitic loads in organs, it is particularly interesting that significantly lower parasitic loads were observed in the brain. Recent reports have elucidated that the processes leading to DC migration into the brain parenchyma in the context of toxoplasmic encephalitis are complex and include signaling through multiple chemokine receptors [40]. The present study does not address the passage of the parasite across the blood brain barrier and whether the infected DC directly transport parasites into the CNS. Alternative but not mutually exclusive hypotheses are possible for the observed differences in parasitic loads in the brain. First, GABAergic inhibition of DC, similar to pertussis toxin treatment [14], [40], may reduce the number of DC that reach the CNS microvasculature. Second, intracellular localization, e.g. in migratory DC, may offer a safe intracellular niche *per se* for targeted delivery to organs [4], [18]. Third, differential transfer of the parasite to other immune cells, e.g. NK, T cells, during infection may modulate the dissemination and passage of *T. gondii* and of infected leukocytes to the CNS [45]–[47]. The relative contribution of these processes to the passage of *T. gondii* across the blood brain barrier remains unknown. Nonetheless, adoptive transfers with infected DC led to higher parasitic loads in the CNS in models of toxoplasmosis and neosporosis [14], [16], [18], while adoptive transfer of infected macrophages and lymphocytes did not [36]. Different results have also been reported on the contribution of CD11b^+^/monocytic cells [16], [48]. Additionally, these processes need to be tested in the context of oral natural infections in future research.

On the technical note, bioluminescence imaging of the brain *ex vivo* failed to detect significant differences between groups treated with GABAergic inhibitors and untreated groups beyond day 2 post-infection. This could be due to the overall low parasitic loads in the brain and to the relatively high variations between individual mice, as demonstrated by plaquing assays. It also indicates that plaquing assays remain the superior method in detecting and quantifying viable parasitic loads in the CNS [30].

The effects of GABA on the immune system have not been extensively studied. In human blood, GABA_A_ receptor subunits haven been detected in CD4^+^ and CD8^+^ T cells, B cells and certain monocytes [35], [49]. In rodents, functional GABA_A_ receptors have been described in T cells [50] and peritoneal macrophages [51]. Activation of GABA_A_ receptors has been shown to inhibit T cell proliferation [50], [52], [53] and autoimmune inflammation [51], [54]. Thus, it is conceivable that GABA release by *T. gondii*-infected DC may modulate DC activation to prevent T-cell proliferation during the early phase of infection.

The ‘inflammatory reflex’ is a concept gaining ground in the field of immunology in demonstrating that neurotransmitters can interact and influence immune cell function [55]. This is an interesting perspective given the psychobehavioural impact of chronic *Toxoplasma* infection in humans and rodents [56]. Also similar to DC, microglia respond with a migratory phenotype upon *T. gondii* infection [47] and their migratory behavior in the CNS could be modulated by ambient GABA. In fact GABA has been shown to suppress the reactivity of microglia [57], leading to attenuation of IL-6 and IL-12 responses [58]. Furthermore, GABA levels in the CNS range from submicromolar outside of synapses to millimolar concentrations in active synapses [59], [60] whilst GABA is present in peripheral tissues at around 100 nM [61]. Here, the current responses to GABA by DC exhibited characteristics of neuronal synaptic and extrasynaptic GABA-activated currents [62]. The synaptic-like currents responded rapidly and then decayed whereas the extrasynaptic-like currents activated with a delay and maintained low current amplitudes for an extended period. The finding that 1 µM GABA concentrations gave rise to robust transient phasic and tonic currents confirms that physiological GABA concentrations may be sufficient to activate both types of currents in DC. This also raises the prospect of GABA acting as a chemoattractant to mitigate ‘homing’ of the infected leukocytes to the CNS and as a possible modulator of microglia-mediated parasite dissemination in the CNS [47]. Human cord blood-derived hematopoietic and progenitor cells have been reported to migrate towards a GABA gradient [63]. In contrast, our *in vitro* chemotaxis data using GABA as a chemoattractant suggests that GABA alone is not sufficient to mitigate directional migration of *T. gondii*-infected DC *in vitro*.

A recently proposed model envisages a complex interplay between ambient GABA, GABA_A_ receptor activation, and chloride transport as regulators of interneuron migration [64]. It is conceivable that the DC GABAergic system may be working in a similar manner in relation to the hypermigratory phenotype exhibited by *T. gondii*-infected DC. Our model comprises: 1) DC invasion by *Toxoplasma*, resulting in increased GABA production; 2) GABA is shuttled out of the cell by GABA transporters, leading to an autocrine effect in activating GABA_A_ receptors; 3) chloride ion efflux by GABA_A_ receptor channels and subsequent calcium influx maintain DC in a depolarizing migratory state. The effector mechanisms leading to increased GABA production in DC as a consequence of *T. gondii*-infection await further investigation. It has been shown that *T. gondii*-infection can lead to extensive transcriptional regulation of host genes in DC [65] and a modulated transcription of GAT4 and of GABA_A_ α_3_, β_3_ and ρ_1_ subunits was observed in infected DC. We found no evidence of significant production of GABA by extracellular parasites but a modulation of the DC biosynthesis of GABA by intracellular parasites cannot be excluded.

In summary, we provide substantial evidence that the DC GABAergic system plays a significant role in the maintenance of the *T. gondii*-induced hypermigratory phenotype observed in infected DC. To the best of our knowledge, this constitutes the first report showing that the GABAergic system can be utilized by an intracellular pathogen to modulate host cell motility and potentiate systemic dissemination. It remains to be seen whether other pathogens also utilize the GABAergic system to facilitate the establishment of an infection. Further investigation of the specific molecules and pathways involved will enable a greater understanding of the diverse roles that the GABAergic system may play outside the CNS.

## Materials and Methods

### Ethics statement

All protocols involving animals were approved by the Regional Animal Research Ethical Board, Stockholm, Sweden, following proceedings described in EU legislation (Council Directive 86/609/EEC). The Regional Ethics Committee, Stockholm, Sweden, approved protocols involving human cells. All donors received written and oral information upon donation of blood at the Karolinska University Hospital. Written consent was obtained for utilization of white blood cells for research purposes. The ethics committees approved this consent procedure.

### Parasites and cell lines

Tachyzoites from the green fluorescence protein (GFP) and luciferase-expressing *T. gondii* line PTGluc (type II, cloned from ME49/PTG-GFPS65T) [30], RH-LDMluc (Type I, cloned from RH-GFPS65T) [30], CTGluc (type III) [66] and PRU-RFP [67] were maintained by serial 2-days passage in human foreskin fibroblast (HFF) monolayers. HFFs were propagated in Dulbecco's modified Eagle's medium (DMEM; Invitrogen) with 10% fetal bovine serum (FBS), gentamicin (20 µg/ml, Gibco), glutamine (2 mM, Gibco) and HEPES (0.01 M, Gibco) referred to as complete medium (CM).

### Mice

C57BL/6 mice (6–10 weeks old) were maintained and bred at the animal facility of the department of Microbiology, Tumor and Cell Biology, Karolinska Institutet (Stockholm, Sweden). For bioluminescence assays, male BALB/c mice (6–8 weeks old) were purchased from Charles River (Sulzfeld, Germany) and maintained under pathogen-free conditions.

### *In vitro* generated DC, monocytes and astrocytes

Mouse bone marrow-derived DC were generated as described [68]. Briefly, cells from bone marrow of C57BL/6 mice were grown in CM containing 20% supernatant from the GM-CSF-secreting cell line X63 or 10 ng/ml recombinant mouse GM-CSF (Peprotech). Loosely adherent cells were harvested on day 6. To generate human monocyte-derived DC, buffy coats obtained from healthy blood donors at the Karolinska University Hospital Blood Center were treated with 1 ml RosetteSep (StemCell Technologies) per 15 ml of buffy coat, followed by centrifugation on Lymphoprep (Axis.Shield PoC AS) gradients. The population, defined as monocytes, exhibited CD14^+^ (DakoCytomation) and <1% CD3^+^/19^+^ (BD Biosciences) as evaluated by flow cytometry (FACSCalibur, BD Biosciences). DC were generated as described previously [69]. Briefly, purified cells were cultured 7 days in CM supplemented with 100 ng/ml GM-CSF (Peprotech) and 12.5 ng/ml IL-4 (R&D Systems). DC were typified by expression of CD1a, CD11b, CD14 (DakoCytomation), CD80, CD83, CD86, HLA-DR, CD11a, CD18 (BD Biosciences). Primary astrocytes were generated from cortices from 1–3 day-old C57BL/6 mice as previously described [47].

### Immunocytochemistry

Human monocyte-derived DC were cultured on poly-L-lysine-coated glass coverslips (Sigma) and challenged with freshly egressed tachyzoites (PTGluc, MOI 1) for 16 h. After fixation (3% paraformaldehyde; Sigma) and blockade (5% FBS; Gibco), the cells were stained with mouse-anti human β_3_ (NeuroMab; clone N87/25; UC Davis/NIH NeuroMab Facility; 1∶250). Anti-mouse Alexa Fluor-conjugate (Invitrogen) was used as secondary antibody. Slides were mounted using VectaShield with DAPI (Vector Laboratories) and assessed by epifluorescence microscopy (Leica DMRB).

### Methods for electrophysiology

DC (3×10^6^ cells) were collected and centrifuged for 2 min at 100 *g*. The supernatant was removed, the pellet washed with the extracellular solution, and centrifuged for 2 min at 100 *g*. The pellet was then resuspended using 100 µl of the extracellular solution. Nanion's Port-a-Patch chip technology (Nanion, Germany) was used to voltage-clamp the cells. A cell suspension of 5 µl was dispensed into the extracellular chamber containing the recording chip of 2–3.5 MΩ resistance. The whole-cell configuration was established and currents were recorded at holding potential of −80 mV. GABA (1 µM and 1 mM) or GABA plus 100 µM SR95531 (GABA_A_ antagonist, Sigma-Aldrich) were prepared with the extracellular recording solution and perfused into the extracellular chamber at a rate of 1 ml/min. Extracellular solution in mM: 145 NaCl , 5 KCl, 1 MgCl_2_, 1.8 CaCl_2_ , 10 TES (pH 7.3) and 297 mOsm/kg. Internal recording solution in mM: 50 CsCl , 10 NaCl, 60 Cs-Fluoride, 20 EGTA, 10 TES (pH 7.3) and 284 mOsm/kg. Extracellular recording solution in mM: 80 NaCl, 3 KCl, 10 MgCl_2_, 35 CaCl_2_, 10 HEPES (pH 7.3) and 296 mOsm/kg. All patch-clamp recordings were performed at room temperature (20–22°C). Patch-clamp recordings were done using an Axopatch 200B amplifier, filtered at 2 kHz, digitized on-line at 10 kHz using an analogue-to-digital converter and analyzed with pClamp software (Molecular Devices, USA).

### RNA analysis with real time qPCR

Total RNA was extracted using TRIzol reagent (Invitrogen). First-strand cDNA was synthesized using Superscript II Reverse Transcriptase (Invitrogen). All primers were initially screened for signal detection using cDNA and conventional PCR. Following sequence confirmation, gene transcription levels were monitored. Using the SYBR Green Master Mix (Applied Biosystems) PCR reactions were carried out on the Applied Biosystems 7500 Fast Real-Time PCR System using the following program: 10 min holding at 95°C, 40 cycles of 95°C for 15 s, 60°C for 1 min. A dissociation stage (95°C for 15 s, 60°C for 1 min, 95°C for 15 s) was added to generate a melting curve for data analysis. Glyceraldehyde 3-phosphate dehydrogenase (GAPDH) was used as the reference gene. The data was analyzed using Sequence Detection Software v.1.3.1 (Applied Biosystems) and fold changes in expression were calculated using the comparative ΔCT method against the non-infected DC control. Primers against the 19 GABA_A_R subunits (Table S1) were designed using OligoPerfect Designer software (Invitrogen) and Primer-BLAST (http://www.ncbi.nlm.nih.gov/tools/primer-blast/), and validated using OligoAnalyzer (http://eu.idtdna.com/analyzer/Applications/OligoAnalyzer). The primers were purchased from Invitrogen or Qiagen.

### GABA and glutamate ELISA

DC were plated at a density of 1×10^6^ cells per well and incubated with freshly egressed *T. gondii* tachyzoites (MOI 1) or with soluble extracts and reagents in a total volume of 700 µl CM for 24 h. Freshly egressed tachyzoites were heat-inactivated at 56°C for 30 min. Parasite lysates were obtained by harvesting supernatants after sonicating 1×10^6^ tachyzoites for three 30 s pulses (Soniprep 150). Reagents were purchased from Sigma-Aldrich unless stated otherwise, and added at the following final concentrations: Semicarbazide (SC, 50 µM); (S)-SNAP-5114 (SNAP; 50 µM); LPS (100 ng/ml). The cells were centrifuged at 4000 *g* for 2 min, and the medium supernatant collected and stored at -70°C until analysis. Samples were extracted, derivatized, and incubated with antiserum according to manufactures protocol (GABA or Glutamate Research ELISA kits, Labor Diagnostica Nord, Nordhorn, Germany). GABA and glutamate concentrations were quantitatively determined by ELISA, monitored at 450 nm (Multiskan EX, Labsystem Oy, Finland).

### *In vitro* migration assays and replication assays

DC and monocytes were plated at a density of 1×10^6^ cells per well and incubated with freshly egressed *T. gondii* tachyzoites (at indicated MOI) or with soluble extracts and reagents in CM for 6 h. Cells were then gently transferred into triplicate transwell filters (8 µm pore size; BD) at a density of 2×10^5^ per well, and incubated as indicated. Reagents were added at the following final concentrations: Muscimol (300 µM); Bicuculline (50 µM); Baclofen (500 µM); CGP35348 (500 µM); Semicarbazide (50 µM); SNAP 5114 (50 µM); LPS (100 ng/ml); GABA (0.5 µM). When indicated, parasite viability and replication was assessed by plaquing assays on HFF monolayers and vacuole counts in DC as previously described [47]. DC integrity was monitored by propidium iodide staining. In the GAD/GAT4 migration restoration experiment, GABA supernatant was obtained from infected DC which had been incubated for 24 h in CM to enrich for DC derived GABA or commercially available exogenous GABA was added to the transwell medium following DC transfer. Migrated DC were quantified using a neubauer hemocytometer.

### Chemotaxis assays, imaging and analysis

Non-infected DC or *Toxoplasma*-infected DC (PTGluc/MOI 1) were incubated ± LPS (200 ng/ml) as well as ± SNAP5114 (50 µM) or Semicarbazide (50 µM) for 24 h. Following incubation, DC were mixed with Collagen I solution (3 mg/ml, Gibco), 7.5% NaHCO_3_ (Invitrogen) and 10× Minimum Essential Medium (MEM; Invitrogen). Approximately 7.5×10^4^ cells were loaded into μ-slide 3D chemotaxis chamber slides (Ibidi) and placed at 37°C, 5% CO_2_ to allow gel formation. Then, to establish a gradient, 1.25 µg/ml CCL19 (R&D systems) or GABA (0.5 µM or 5 µM) were added to one chamber reservoir whilst the other reservoir was filled with CM. Control experiments used CM in both reservoirs. Cell migration was monitored using a Zeiss AxioImager Z1 microscope and AxioVision software (version 4.7.2). Images were taken every 60 s for 60 min. Cell tracking and chemotaxis analysis were performed using ImageJ (http://imagej.nih.gov/ij/) with Manual Tracking (Cordelières, Institute Curie) and Chemotaxis Tool (Ibidi) plugins.

### Flow cytometry

CCR7 expression of human monocyte-derived DC was studied using FITC-labeled anti-CCR7 and mouse IgG2a isotype control antibodies (R&D Systems). Murine bone marrow-derived DC were stained with CCL19-Fc (eBiosciences), CD 40, CD80, CD86, MHC II, CD18 antibodies (BD Biosciences) as indicated by the manufacturer. Data were generated using a CyAn ADP (Beckman Coulter) or a FACSCalibur (BD Bioscience) flow cytometer. Fluorescence-activated sorting of *Toxoplasma*-infected cells was performed at the Center for Cell Analysis, Karolinska institutet on a FACSAria (BD Bioscience) system. Dead cells were gated out by SYTOX Blue stain (Invitrogen) and pre-sorting infection rates were 43–63%. Data analysis was done with FACSDiva software, version 6.1.3 (BD Bioscience).

### Adoptive transfers of DC

DC were challenged with freshly egressed PTGluc tachyzoites for 6 h at MOI 1. Extracellular parasites were removed following three washes at 80 *g*. Following infection and resuspension in PBS, 5×10^4^ colony forming units (cfu) were adoptively transferred into male BALB/c mice. Total numbers of cfu injected into animals was confirmed by plaquing assays [70]. SNAP 5114 (50 µM) and Semicarbazide (50 µM) was added upon DC infection for 5 h. When indicated, such groups were treated with an additional 50 µM combination therapy of SNAP 5114 and Semicarbazide for 1 h, and added to PBS DC suspension prior to injection.

DC were stained with 5 µM 5-,6-(4-chloromethyl)benzoyl-amino-tetramethylrhodamine (CMTMR; Molecular Probes) or 5 µM carboxyfluorescein diacetate, succinimidyl ester (CFSE; Invitrogen) as indicated by the manufacturer. Stained cells (±50 µM SC+50 µM SNAP for 6 h) were then injected i.p. into BALB/c mice. After 12 h, the spleens were harvested, homogenized and cells were analyzed by flow cytometry (FACScalibur). An intraperitoneal lavage was performed and cells were similarly analyzed by flow cytometry.

### *In vivo* bioluminescence imaging (BLI)

BLI was performed as described [30]. Briefly, BALB/c mice inoculated i.p. with freshly egressed PTGluc tachyzoites, or with PTGluc-infected DC ± Semicarbazide and SNAP 5114 were injected i.p. with 1.5 mg D-luciferin potassium salt (Caliper Life Sciences, Hopkinton, MA, USA) and anaesthetized with 2.3% isoflurane prior to BLI. Ten min after injection of D-luciferin, biophotonic images were acquired at a binning of 8 (medium) for 180 s with an In Vivo Imaging System Spectrum CT (Caliper Life Sciences). For *ex vivo* imaging of organs, mice were injected i.p. with 1.5 mg D-luciferin and euthanized after 10–15 min. Organs were extracted as assessed as above. Analysis of images and assessment of photons emitted from a region of interest (ROI) was performed with Living Image software (version 4.2; Caliper Life Sciences).

### Plaquing assays

Plaquing assays were performed as described [14]. Briefly, organs were extracted and homogenized under conditions that did not affect parasite viability. The number of parasites was determined by plaque formation on HFF monolayers.

### Statistical analysis

All statistics were performed using Minitab version 15 (Minitab Inc, PA, USA).

### Online supplemental material

Table S1 shows the 19 subunit primer pair sequences used to screen DC and astrocyte cDNA for GABA_A_R subunit transcripts and primer sequences for GAD 65, GAD 67, GAT4 and GAPDH cDNA. Figure S1 shows the ELISA-determined glutamate levels from non-infected and *Toxoplasma*-infected DC supernatant. Figure S2 shows GABA secretion and transmigration by monocytes upon challenge with *T. gondii.* Figure S3 shows GABA secretion of DC challenged with *T. gondii* type I, II and III strains. Figure S4 shows immunocytochemistry of infected and non-infected DC. Figure S5 shows parasite replication in DC in the presence of GABAergic inhibitors. Figure S6 shows the effects of GABAergic inhibitors on tachyzoite-infected DC and extracellular tachyzoites. Figure S7 shows effects of GABAergic inhibition on activation and maturation markers of DC. Figure S8 shows flow cytometric analyses of DC *in vivo* after GABAergic inhibition.

## Supporting Information

Figure S1 **DC glutamate secretion is transiently affected by** ***Toxoplasma*** **infection.** Mouse DC were challenged with PTGluc tachyzoites (MOI 1) and glutamate levels were analyzed at indicated time points using a glutamate ELISA kit as described in Materials and Methods. Values represent means (±SEM) from two experiments performed in quadruplicate. (*) indicates a statistically significant increase in glutamate levels following infection at 3 h (P = 0.034) and 8 h (P<0.001). However, there were no significant differences in glutamate levels by the end of the time course (GLM ANOVA, Tukeys pairwise comparison, P>0.05).
(TIF)

Figure S2 **GABA secretion and transmigration by monocytes upon challenge with** ***T. gondii.*** (**A**) Monocytes respond with GABA secretion upon challenge with *T. gondii*. Freshly isolated human monocytes from four donors (D1–D4) were incubated with PTGluc tachyzoites (MOI 1) for 24 h. GABA in the supernatant was quantitatively determined by ELISA as described in Materials and Methods. For each donor, values represent mean (±SD) performed in triplicate. (**B**) Transmigration of freshly isolated monocytes. Monocytes were challenged with PTGluc tachyzoites (MOI 3) or PTGluc tachyzoites plus SNAP (GAT4 inhibitor, 50 µM) and SC (GAD inhibitor, 50 µM) as described in Materials and Methods. Monocytes were then transferred to transwells, and allowed to transmigrate in the presence of CM (complete medium) or inhibitors (SNAP, 50 µM + SC, 50 µM). Cell migration was determined using a neubauer hemocytometer. Values represent means (±SD) from two different human donors done in triplicate. (*) indicates significant difference (P<0.05, Student's t-test). ns: non-significant.
(TIF)

Figure S3 **GABA secretion of DC challenged with** ***T. gondii*** **type I, II and III strains.** Mouse DC were challenged with RH-LDMluc (type I), PTGluc (type II) or CTGluc (type III) at indicated MOIs. GABA in the supernatant was quantitatively determined after 24 h by ELISA as described under Materials and Methods. One representative experiment performed in duplicate is shown. Performed twice with similar result. Non-significant differences were observed between strains (P>0.05, Student's t-test).
(TIF)

Figure S4 **Infected and non-infected DC express the GABA_A_ receptor β_3_ subunit.** Micrographs show immunocytochemistry of DC challenged with GFP-expressing *T. gondii* tachyzoites (PTGluc, green) stained with GABA_A_ receptor β_3_ subunit monoclonal antibody (red) and DAPI (blue). Stainings were performed as indicated under Materials and Methods. Scale bar: 10 µm.
(TIF)

Figure S5 **Parasite replication in the presence of GABAergic inhibition of DC.** Replication of tachyzoites in DC assessed by vacuole size counts at the indicated time points as described in Materials and Methods. Non-significant differences were observed between samples in complete medium (CM) and samples treated with a combination of SC (GAD inhibitor, 50 µM) and SNAP (GAT4 inhibitor, 50 µM) (*P*>0.05; Mann-Whitney U test). Geneticin, a blocker of polypeptide synthesis in eukaryotic cells, was used as a reference control. Significant differences were observed in samples treated with geneticin (*P*<0.05; Mann-Whitney U test).
(TIF)

Figure S6 **GABAergic inhibitors target host cell-derived GABA production and transport.** Extracellular PTGluc tachyzoites (Toxo) or *tachyzoite*-infected mouse DC (MOI 1; Toxo-DC) were incubated for 24 h in CM under different conditions. SC (GAD inhibitor, 50 µM); SNAP (GAT4 inhibitor, 50 µM). GABA levels were analyzed using a GABA ELISA kit as described in Materials and Methods. Values represent means (±SE) from two independent experiments performed in quadruplicate. (*) indicates a significant decrease in GABA production compared to untreated *Toxoplasma*-infected DC (P<0.008, Mann Whitney U test).
(TIF)

Figure S7 **Co-stimulatory molecule stainings and maturation markers of DC in presence of GABAergic inhibitors.** Mouse bone marrow-derived DC were treated as indicated for 12–16 h and stained as described under Materials and Methods. “Untreated” and “LPS” indicate DC in complete medium or exposed to LPS (200 ng/ml), respectively; “Toxo” indicates DC challenged with freshly egressed GFP-expressing PTGluc tachyzoites (MOI 1). “SC + SNAP” indicates treatment with SC (GAD inhibitor, 50 µM) and SNAP (GAT4 inhibitor, 50 µM). Results are representative of two or three independents experiments with similar results.
(TIF)

Figure S8 **Prevalence and dissemination of adoptively transferred DC upon GABAergic inhibition.** BALB/c mice were inoculated i.p with 5×10^6^ untreated DC labeled with CMTMR and 5×10^6^ untreated DC labeled with CFSE (top panel) or with 5×10^6^ untreated CMTMR-labeled DC and 5×10^6^ SC+SNAP treated DC, labeled with CFSE (bottom panel). After 12 h, an intraperitoneal lavage was performed and spleens were extracted and processed for flow cytometry as indicated in Materials and Methods. Dot plots of CMTMR (y-axis) and CFSE (x-axis) show numbers of SC+SNAP treated DC as compared to the untreated population, in both the spleen and peritoneal lavage. Numbers indicate the relative portion of each quadrant related to the total population. Double-positive (CMTMR/CFSE) cells may indicate uptake in phagocytic cells. Data shown are from one experiment, and are representative of the results from 2 independent experiments.
(TIF)

Table S1 **Primer pair sequences used to screen DC and astrocyte cDNA for 19 GABA_A_R subunit transcripts and primer sequences for GAD 65, GAD 67, GAT4 and GAPDH cDNA. **Primers were designed as indicated under Materials and Methods **.**
(DOCX)
