# Supplementary figures and images for: GABAergic Signaling Is Linked to a Hypermigratory Phenotype in Dendritic Cells Infected by Toxoplasma gondii
Source: PLoS Pathog. 2012 Dec 6;8(12):e1003051. doi: 10.1371/journal.ppat.1003051 (PMC3516538; doi:10.1371/journal.ppat.1003051)

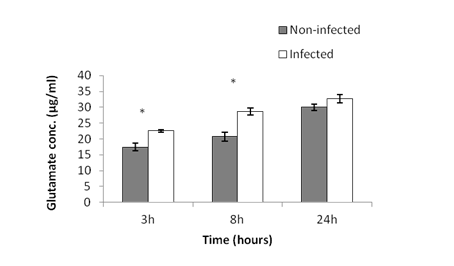

Supplement: Figure S1 — DC glutamate secretion is transiently affected by Toxoplasma infection. Mouse DC were challenged with PTGluc tachyzoites (MOI 1) and glutamate levels were analyzed at indicated time points using a glutamate ELISA kit as described in Materials and Methods. Values represent means (±SEM) from two experiments performed in quadruplicate. (*) indicates a statistically significant increase in glutamate levels following infection at 3 h (P = 0.034) and 8 h (P<0.001). However, there were no significant differences in glutamate levels by the end of the time course (GLM ANOVA, Tukeys pairwise comparison, P>0.05). (TIF) [file ppat.1003051.s001.tif]

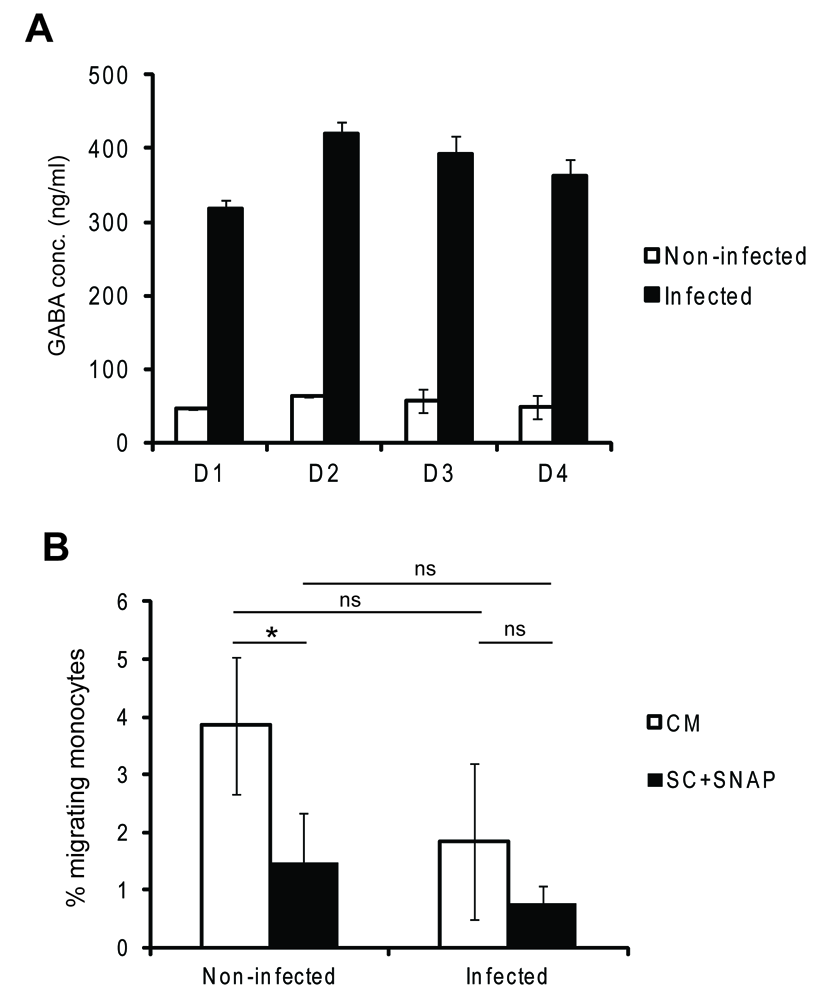

Supplement: Figure S2 — GABA secretion and transmigration by monocytes upon challenge with T. gondii. (A) Monocytes respond with GABA secretion upon challenge with T. gondii. Freshly isolated human monocytes from four donors (D1–D4) were incubated with PTGluc tachyzoites (MOI 1) for 24 h. GABA in the supernatant was quantitatively determined by ELISA as described in Materials and Methods. For each donor, values represent mean (±SD) performed in triplicate. (B) Transmigration of freshly isolated monocytes. Monocytes were challenged with PTGluc tachyzoites (MOI 3) or PTGluc tachyzoites plus SNAP (GAT4 inhibitor, 50 µM) and SC (GAD inhibitor, 50 µM) as described in Materials and Methods. Monocytes were then transferred to transwells, and allowed to transmigrate in the presence of CM (complete medium) or inhibitors (SNAP, 50 µM + SC, 50 µM). Cell migration was determined using a neubauer hemocytometer. Values represent means (±SD) from two different human donors done in triplicate. (*) indicates significant difference (P<0.05, Student's t-test). ns: non-significant. (TIF) [file ppat.1003051.s002.tif]

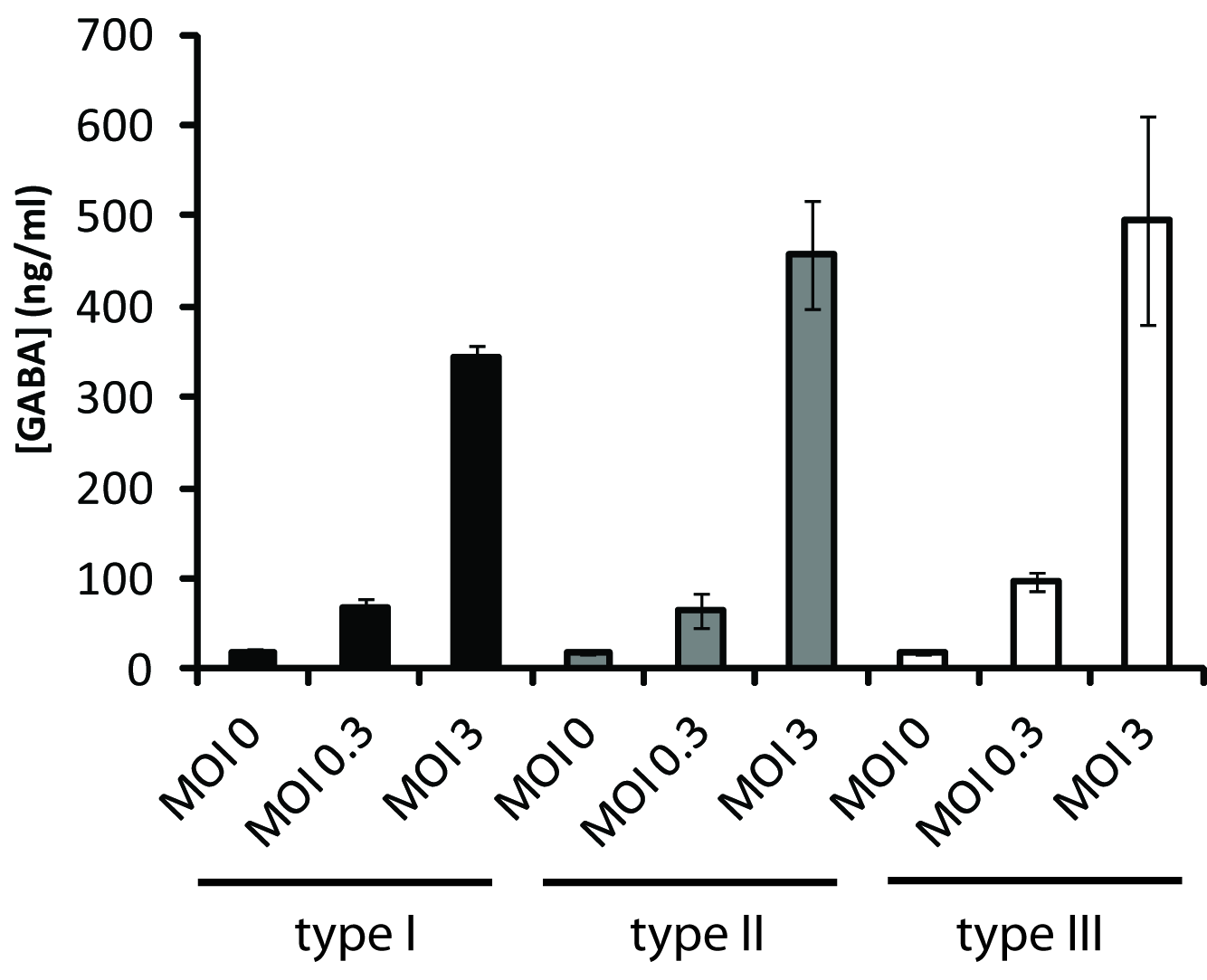

Supplement: Figure S3 — GABA secretion of DC challenged with T. gondii type I, II and III strains. Mouse DC were challenged with RH-LDMluc (type I), PTGluc (type II) or CTGluc (type III) at indicated MOIs. GABA in the supernatant was quantitatively determined after 24 h by ELISA as described under Materials and Methods. One representative experiment performed in duplicate is shown. Performed twice with similar result. Non-significant differences were observed between strains (P>0.05, Student's t-test). (TIF) [file ppat.1003051.s003.tif]

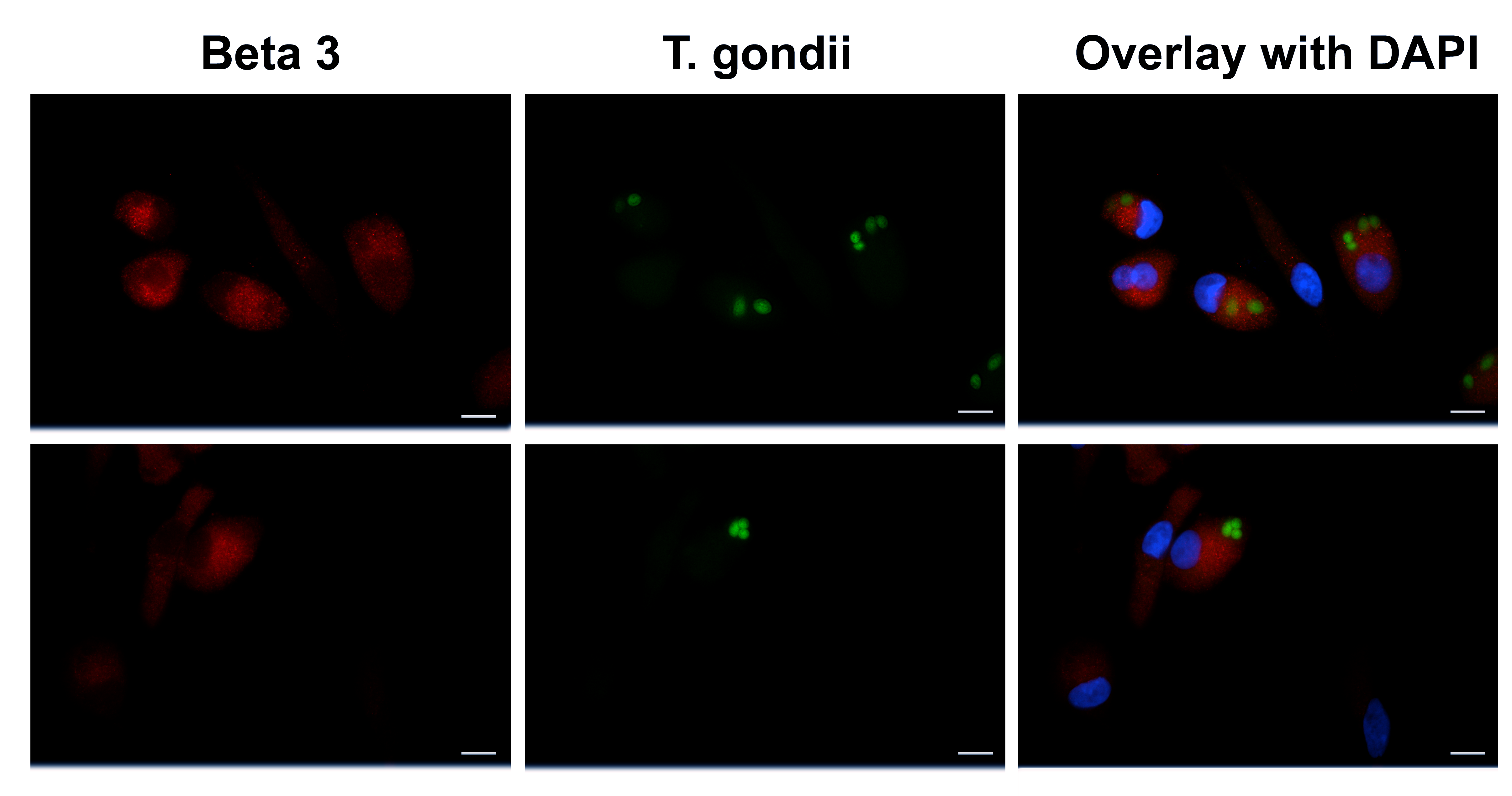

Supplement: Figure S4 — Infected and non-infected DC express the GABAA receptor β3 subunit. Micrographs show immunocytochemistry of DC challenged with GFP-expressing T. gondii tachyzoites (PTGluc, green) stained with GABAA receptor β3 subunit monoclonal antibody (red) and DAPI (blue). Stainings were performed as indicated under Materials and Methods. Scale bar: 10 µm. (TIF) [file ppat.1003051.s004.tif]

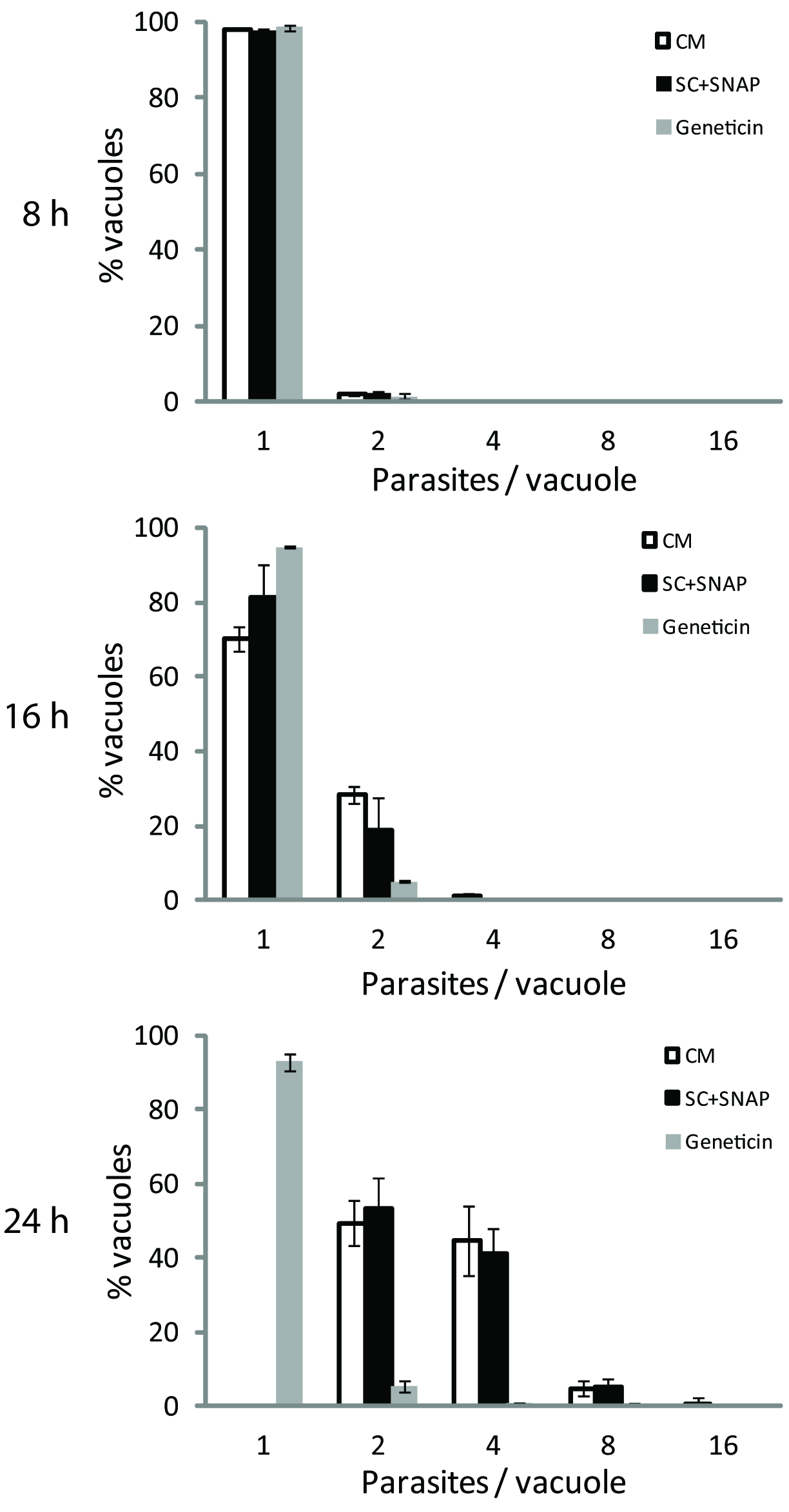

Supplement: Figure S5 — Parasite replication in the presence of GABAergic inhibition of DC. Replication of tachyzoites in DC assessed by vacuole size counts at the indicated time points as described in Materials and Methods. Non-significant differences were observed between samples in complete medium (CM) and samples treated with a combination of SC (GAD inhibitor, 50 µM) and SNAP (GAT4 inhibitor, 50 µM) (P>0.05; Mann-Whitney U test). Geneticin, a blocker of polypeptide synthesis in eukaryotic cells, was used as a reference control. Significant differences were observed in samples treated with geneticin (P<0.05; Mann-Whitney U test). (TIF) [file ppat.1003051.s005.tif]

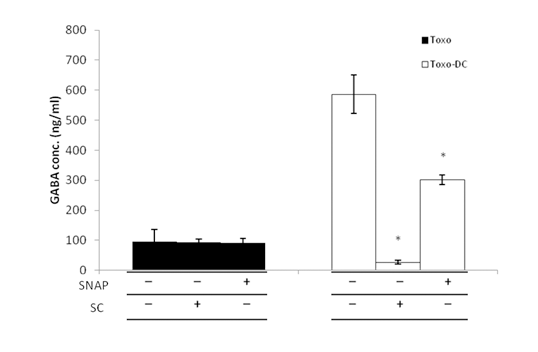

Supplement: Figure S6 — GABAergic inhibitors target host cell-derived GABA production and transport. Extracellular PTGluc tachyzoites (Toxo) or tachyzoite-infected mouse DC (MOI 1; Toxo-DC) were incubated for 24 h in CM under different conditions. SC (GAD inhibitor, 50 µM); SNAP (GAT4 inhibitor, 50 µM). GABA levels were analyzed using a GABA ELISA kit as described in Materials and Methods. Values represent means (±SE) from two independent experiments performed in quadruplicate. (*) indicates a significant decrease in GABA production compared to untreated Toxoplasma-infected DC (P<0.008, Mann Whitney U test). (TIF) [file ppat.1003051.s006.tif]

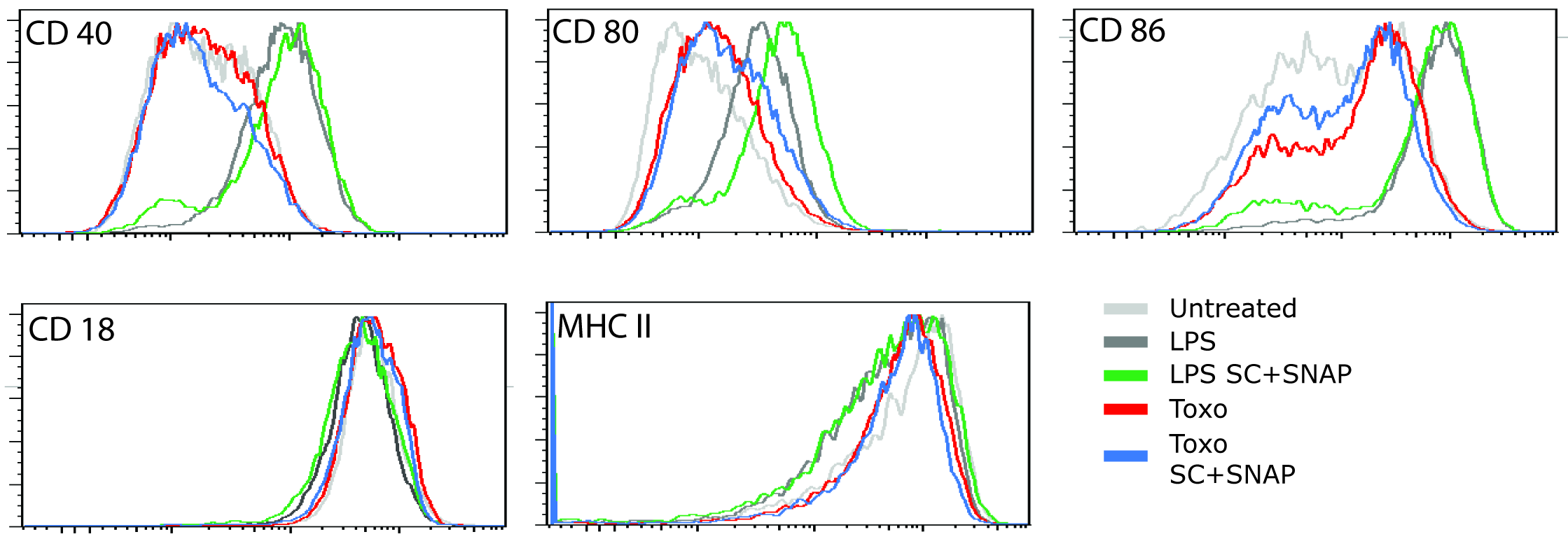

Supplement: Figure S7 — Co-stimulatory molecule stainings and maturation markers of DC in presence of GABAergic inhibitors. Mouse bone marrow-derived DC were treated as indicated for 12–16 h and stained as described under Materials and Methods. “Untreated” and “LPS” indicate DC in complete medium or exposed to LPS (200 ng/ml), respectively; “Toxo” indicates DC challenged with freshly egressed GFP-expressing PTGluc tachyzoites (MOI 1). “SC + SNAP” indicates treatment with SC (GAD inhibitor, 50 µM) and SNAP (GAT4 inhibitor, 50 µM). Results are representative of two or three independents experiments with similar results. (TIF) [file ppat.1003051.s007.tif]

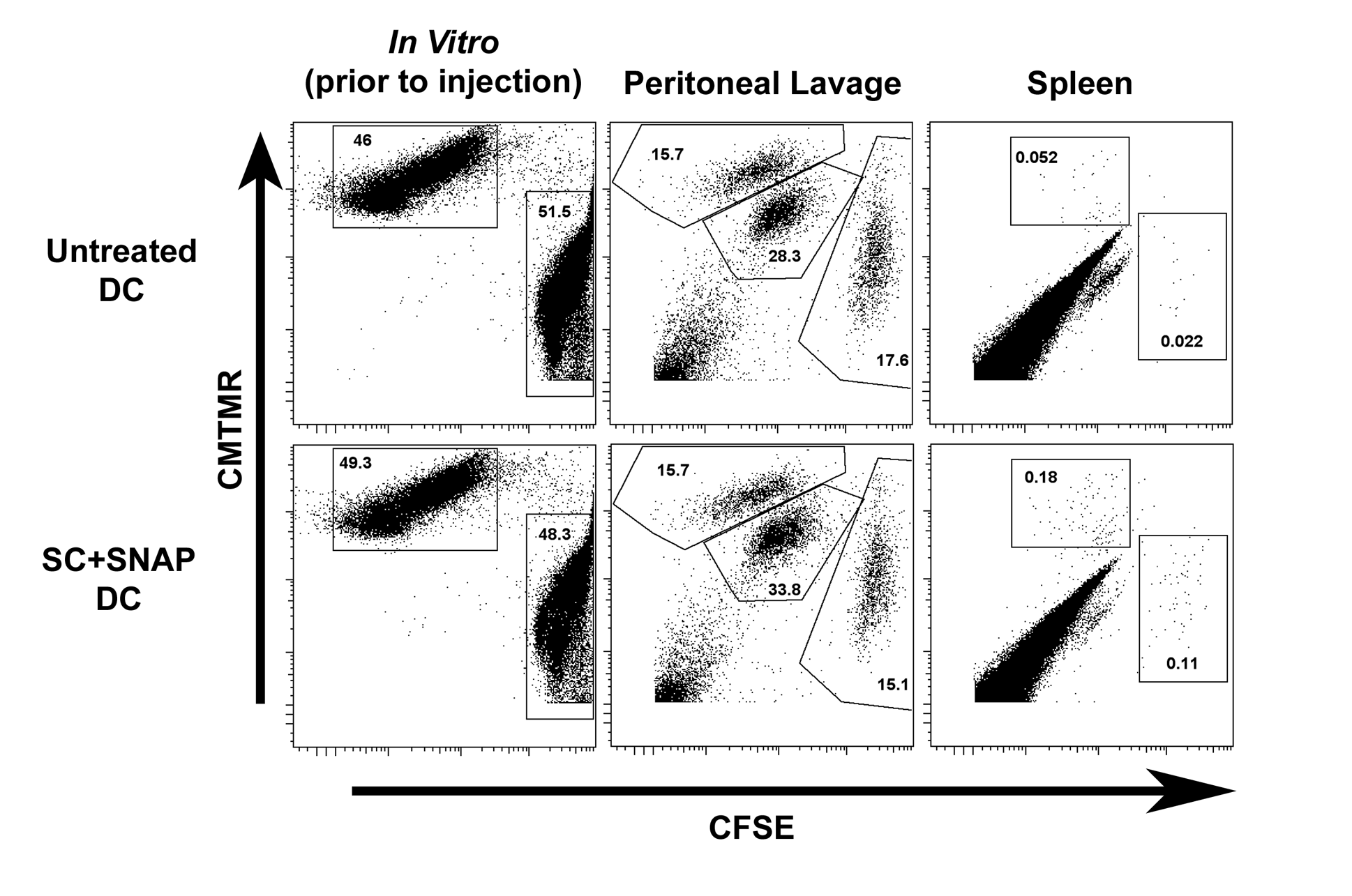

Supplement: Figure S8 — Prevalence and dissemination of adoptively transferred DC upon GABAergic inhibition. BALB/c mice were inoculated i.p with 5×10^6^ untreated DC labeled with CMTMR and 5×10^6^ untreated DC labeled with CFSE (top panel) or with 5×10^6^ untreated CMTMR-labeled DC and 5×10^6^ SC+SNAP treated DC, labeled with CFSE (bottom panel). After 12 h, an intraperitoneal lavage was performed and spleens were extracted and processed for flow cytometry as indicated in Materials and Methods. Dot plots of CMTMR (y-axis) and CFSE (x-axis) show numbers of SC+SNAP treated DC as compared to the untreated population, in both the spleen and peritoneal lavage. Numbers indicate the relative portion of each quadrant related to the total population. Double-positive (CMTMR/CFSE) cells may indicate uptake in phagocytic cells. Data shown are from one experiment, and are representative of the results from 2 independent experiments. (TIF) [file ppat.1003051.s008.tif]
