# Supplementary material for: GABAergic Signaling Is Linked to a Hypermigratory Phenotype in Dendritic Cells Infected by Toxoplasma gondii
Source: PLoS Pathog. 2012 Dec 6;8(12):e1003051. doi: 10.1371/journal.ppat.1003051 (PMC3516538; doi:10.1371/journal.ppat.1003051)
Supplement: Table S1 — Primer pair sequences used to screen DC and astrocyte cDNA for 19 GABAAR subunit transcripts and primer sequences for GAD 65, GAD 67, GAT4 and GAPDH cDNA. Primers were designed as indicated under Materials and Methods . (DOCX) [file ppat.1003051.s009.docx]

**Supplementary Table SI.** Primers used to amplify GABA_A_R channel sub-units, GAD 65, GAD 67 and GAPDH cDNA.

| **GABA_A_R Subunit** | **Primer sequence^a^** | **Amplicon size (bp)** | **Amplicon position** |
| --- | --- | --- | --- |
| α_1_ | F:5’-AGTGGTTGTAGCAGAAGATG-3’  R: 5’-AGGAGACTTGGGAGAGAATA-3’ | 200 | 1392-1591 |
| α_2_ | F: 5’-CCAAGTGTCATTCTGGCTGA-3’  R: 5’-CGCATAGGCGTTGTTCTGTA-3’ | 301 | 1095-1395 |
| α_3_ | F: 5’-TCCTGCTGAGACCAAGACCT-3’  R: 5’-GGCTCAATCCAAGCAATGTT-3’ | 361 | 1601-1961 |
| α_3_ RT | F: 5’-TGTCCCTGCCCGCACAGTCT-3’  R: 5’-GGCCGTCGCGTATGCCACTT-3’ | 103 | 1184-1286 |
| α_4_ | F: 5’-TCCCCTGAGTTACAGGTTGG-3’  R: 5’-CAGAAACCATCTTCGCAACA-3’ | 372 | 138-509 |
| α_5_ | F: 5’-GGACTCTTGGATGGCTATGA-3’  R: 5’-ATTGTCAGACGCATGGTGTA-3’ | 344 | 507-850 |
| α_6_ | F: 5’-AACTTTCAGTGGCATTGGAC-3’  R: 5’-GAACCTCCTGGTGTTAGCAA-3’ | 225 | 2133-2357 |
| β_1_ | F: 5’-AAGGTGGAGTTCACAACAGG-3’  R: 5’-TTTGACGTAAGGGATCTTCG-3’ | 249 | 664-912 |
| β_2_ | F: 5’-CACAATGCTTGCCTATGATG-3’  R: 5’-CTTCTCACGGAAGGCTGTAG-3’ | 274 | 1600-1873 |
| β_3_ | F: 5’-CAGTCACCATGGACTGACAC-3’  R: 5’-TGTCTCCTCCCACTGTTACC-3’ | 292 | 3209-3500 |
| γ_1_ | F: 5’-TGTTCAATCTGGTTTACTGG-3’  R: 5’-AGCTAATGAGCAATCTAGGC-3’ | 457 | 1756-2212 |
| γ_2_ | F: 5’-TCTCTGCCCAAGGTCTCCTA-3’  R: 5’-AAGGTGTGTGGCATTGTTCA-3’ | 219 | 959-1177 |
| γ_3_ | F: 5’-CAGGACCTGACAGACAAGCA-3’  R: 5’-TATTGGCAAATGCCTGAACA-3’ | 356 | 1135-1520 |
| δ | F: 5’-GAATCCGTTCCAGACTCAAA-3’  R: 5’-GCACTAGGCTCAACTTCAGG-3’ | 349 | 1339-1687 |
| θ | F: 5’-GCTGGAGGTGGAGAGCTATG-3’  R: 5’-GGAGAGAATGGTGGTGAGGA-3’ | 253 | 614-866 |
| ε | F: 5’-TCAATGCGAAGAACACTTGG-3’  R: 5’-AGAAGTCCAAAGCCGTGAGA-3’ | 327 | 1954-2280 |
| π | F: 5’-CGTCCATGAAGACTTGCTGA-3’  R: 5’-TCAGGCAGGGTAATCCACTC-3’ | 324 | 2294-2617 |
| ρ_1_ | F: 5’-CTGGAAATCGAAAGCTACGC-3’  R: 5’-AGATGTGACGACGCAGAGTG-3’ | 205 | 741-945 |
| ρ_1_ RT | F: 5’-GCTGCTGAGAGCACAGCGCA-3’  R: 5’-TGGGCTGCCTTGCTTGTGGG-3’ | 120 | 159-278 |
| ρ_2_ | F: 5’-CCAAGCCAAGCCATTTGTAT-3’  R: 5’-GTCCCTCCAGTAATGCCTCA-3’ | 227 | 246-472 |
| ρ_3_ | F: 5’-TACTGGAAACACGGCAACAA-3’  R: 5’-ACCGCCTTGACATAGGACAC-3’ | 347 | 634-980 |
| GAD65 | F: 5’-GCTGGAACCACCGTGTATGG-3’  R: 5’-TCCACGTGCATCCAGATCTTAT-3’ | 86 | 1342-1427 |
| GAD67 | F: 5’-TCCACCATCAACGGCATTAT-3’  R: 5’-AGCGGCAGGTGTTGGATAAC-3’ | 88 | 4651-4738 |
| GAPDH | F:5’-CCCATCACCATCTTCCAGCA-3’  R:5’-CGACATACTCAGCACCGGC-3’ | 70 | 277-328 |

^a^ Primers were designed as indicated under Materials and Methods.
